# FuzzyFed-CNN: secure and explainable multimodal federated learning for early Alzheimer’s diagnosis

**DOI:** 10.3389/frai.2026.1852196

**Published:** 2026-06-25

**Authors:** S. Mohanraj, Sujatha Radhakrishnan

**Affiliations:** School of Computer Science Engineering and Information Systems, Vellore Institute of Technology, Vellore, Tamil Nadu, India

**Keywords:** Alzheimer’s disease, clinical metadata, CNN, explainable healthcare models, federated learning, fuzzy features, Grad-CAM, multimodal fusion

## Abstract

**Introduction:**

Alzheimer’s disease (AD) is a progressive neurodegenerative condition that has a great effect on cognitive impairment and quality of life. Timely intervention requires the early and reliable diagnosis of the patient, but current diagnostic systems are frequently troubled with the limitations of data privacy, their lack of interpretability, and the fusion of heterogeneous clinical and imaging data.

**Objective:**

The proposed study suggests FuzzyFed-CNN, an explainable and privacy-oriented multimodal FL system that incorporates CNNs as well as fuzzy inference systems to enhance the early detection of AD and model interpretability and data privacy.

**Methods:**

The suggested framework involves CNN-based extractions of features using the T1-weighted MRI structural scans, and the use of the fuzzy-rule-based reasoning with the demographic and neuropsychological features, such as age, MMSE scores, and hippocampal volume. Experiments were done using a subset of the ADNI and OASIS-3 datasets. The training was conducted in a FL setting using the FedAvg algorithm. The metrics of accuracy, sensitivity, specificity, F1-score, and AUC were used to evaluate model performance.

**Results:**

Experiments that FuzzyFed-CNN with its accuracy, sensitivity, and specificity measure 97.7, 98.0, 99.0, and F1-score of 98.0. The suggested framework was better at performing compared to baseline models such as MobileNet and ResNet., DenseNet, EfficientNet. Grad-CAM visualizations also supported that the model paid attention to clinically significant brain regions, including the hippocampus and the cortical areas.

**Conclusion:**

The results demonstrate that combining multimodal learning, fuzzy reasoning, and federated training can be used to achieve considerable improvements in the diagnosis of AD without damaging patient privacy and improving the interpretability of the models.

## 1 Introduction

AD is a progressive neurodegenerative disease which has a serious impact on cognitive functioning and quality of life. Timely and proper diagnosis is important in the intervention and management of the disease. Recent developments in the area of artificial intelligence have made it possible to use multimodal data (neuroimaging, clinical, and cognitive data) to enhance diagnostic performance (Mohsen, 2025). Nevertheless, the problem of combining heterogeneous sources of data and preserving the privacy and interpretability of the data is very significant (Alzubaidi et al., 2025).

Traditional deep learning architectures, especially Convolutional Neural Networks (CNNs), have been shown to be highly successful in processing MRI scans, yet they, in many cases, do not utilize clinical metadata, which restricts their practicality in the real world (Xiong et al., 2022). Also, the vast majority of multimodal models are based on centralized data storage, which does not align with the rigid privacy laws like the United States Health Insurance Portability and Accountability Act (HIPAA) and the European Union General Data Protection Regulation (GDPR) (Yu et al., 2023). These constraints demonstrate the necessity of a privacy-sensitive, interpretable, and multimodal diagnostic model (Yan et al., 2024).

At the multimodal MRI and clinical feature level, FL enables local training of the data in the present study (Wang et al., 2024). This allows sound generalization in heterogeneous sites (Qi et al., 2023). In that way, two of the most important loopholes can be addressed by FuzzyFed-CNN: (1) how to federate multimodal medical data and remain privacy-friendly, and (2) how to ensure model explainability by compromising diagnostic performance. The proposed framework addresses the area of research related to privacy-preserving and explainable medical AI by incorporating elements such as multimodal learning, fuzzy inference system, and federated learning into an early Alzheimer’s disease diagnosis framework. Timely diagnosis is essential to slow down the process and maximize care. Although deep learning algorithms, and especially CNNs, provide significant results on MRI scans, they do not tend to incorporate clinical features in their context. In addition, the majority of multimodal structures rely on centralized data, which is not consistent with strict laws, including HIPAA and GDPR. These issues point to the necessity of a diagnostic framework that is reliable, understandable, and non-invasive. It has been demonstrated that many CNN-only models are highly accurate on imaging data but do not take into account clinical metadata, limiting their practical application. Current multimodal approaches tend to use centralized data, which poses a risk to privacy and governance. Federated learning (FL) models focus on privacy but seldom incorporate explainability, which restricts trust in clinicians. There are not many studies that have combined fuzzy inference with a deep multimodal model in a federated environment.

The key contributions of the work are as follows:

- Multimodal fusion framework: CNN-based MRI features with fuzzy-rule-based clinical reasoning to improve the accuracy of diagnosis.
- Privacy-preserving federated learning: A FL model that allows institutions to collaborate in learning without sharing raw patient data.
- Explainable AI integration: Use of Grad-CAM and fuzzy inference to improve model transparency and clinical interpretability.
- Strong validation: Full assessment based on ADNI and OASIS datasets, including ablation experiments and control models.

## 2 Related work

### 2.1 CNNs for neuroimaging

Recent advances in artificial intelligence have played a significant role in early neurodegenerative disease detection, and AD has been at the forefront. Magnetic resonance imaging (MRI) neuroimaging has been a key feature of such work because it is able to reveal structural alterations in the brain that give rise to cognitive impairment (Wang et al., 2023). Although the current literature confirms the potential of multimodal learning in detecting Alzheimer’s, some serious concerns arise. First, several CNN-based neuroimaging methods have been reported to be highly accurate but overlook contextual information about the patient, including age, genetic history, or cognitive test results. This kind of narrow focus can potentially lead to technically sound models that are unlikely to be representative of the complexity of actual clinical diagnosis. CNNs can also perform well on MRI scan analysis, capable of learning low-level patterns and spatial hierarchies that are predictive of the course of disease (Wang et al., 2024). However, image-only models will probably miss important context or clinical information such as patient age, genetic risk, or cognitive test scores. Thus, multimodal approaches combining imaging and non-imaging information have been studied to enhance diagnostic accuracy (Eldin, 2026). Our previous research (Mohanraj and Sujatha, 2025) created a CNN–fuzzy–XAI framework for classifying AD using brain MRI scans. This work builds on that by combining FL and multimodal fusion to improve privacy, explainability, and cooperation among institutions for the detection of early-stage AD.

### 2.2 Fuzzy inference in medical AI

Multimodal learning attempts to synthesize multiple sources of information, such as images, numerical biomarkers, and categorical metadata, in the hope of inferring a refined understanding of a patient’s state of health. For Alzheimer’s diagnosis, multimodal models that bridge neuroimaging and carefully crafted clinical data. It improved classification performance and robustness (Jahan et al., 2025). This approach frequently embraces feature-level and decision-level merging. It facilitates complementary strengths across modalities. Fuzzy logic offers a means of expressing uncertainty and imprecision in clinical presentation. This is valuable with the heterogeneity of Alzheimer’s symptomology (Raza et al., 2023). Second, most frameworks have a high level of robustness, but they use centralized data, which poses serious privacy issues, especially when using multimodal models. In reality, there are stringent rules like GDPR and HIPAA, which restrict the sharing of data, and it is not possible to directly apply many of the models that have been found to be effective in research to hospitals. In conjunction with machine learning algorithms, fuzzy systems have demonstrated promise in managing uncertain or overlapping diagnostic features.

### 2.3 Federated multimodal learning

Despite their performance, typical multimodal models require centralized data, which is a critical issue in privacy data and institutional limitations (Cremonesi et al., 2023). FL offers a promising scenario as it can enable the training of the model in decentralized data silos (Bernecker et al., 2022). The approach allows institutions to collectively learn machine learning models without sharing patient information in order to leave the local institution where it would be safe (Zhang et al., 2024). Successful deployments of FL have been made in many healthcare applications to validate the capability of this method to enable real-world clinical cooperation without violating privacy legislations such as GDPR or HIPAA (Zhao et al., 2018). Transparency is a weakness in the previous literature. Although black-box models are accurate, the clinician can hardly trust or verify predictions. This negatively affects clinical acceptance, as clinicians need interpretable evidence that is consistent with well-known medical logic. Some studies have tried to apply fuzzy logic or rule-based reasoning, but not often been applied to deep neural architecture in a federated environment. This is a weakness which points to an opportunity: by integrating explainable reasoning frameworks with privacy-preserving learning, both trust and governance issues may be resolved (AbdelAziz et al., 2024).

Interest is also increasing in using FL on multimodal data. Currently, though, coordinating heterogeneous data structures and learning across institutions renders the training process difficult (Anagnostopoulos et al., 2024). Partial weight sharing, knowledge distillation, or model assembling has been suggested in some recent work to facilitate modality-specific features without compromising collaborative learning (Wang et al., 2022). Taking a look back at the literature, one can see that research has mostly taken technical performance as a priority instead of scalability and ethical deployment. Well-organized, publicly available datasets such as OASIS and ADNI are often used to test models, which may fail to reflect the variability and noise of actual hospital data.

While prior research has built a strong foundation, only a limited number of studies have explored the combined integration of fuzzy inference, multimodal fusion, and federated learning within a single diagnostic framework (Xu et al., 2024). Hence, it fills and expands on current literature by demonstrating how these ingredients together can contribute toward the development of safe, comprehensible, and highly effective diagnostic tools for AD (Huang et al., 2024). As a result, the effective risk is overestimated. One important contribution of the paper is thus to go beyond the performance benchmarking by considering how federated and interpretable approaches can be more responsive to the messy realities of clinical practice. There is a tendency to use federated learning approaches in current research in Alzheimer’s disease because of the need to resolve privacy issues related to multi-institutional datasets in neuroimaging. There were multiple studies showing that federated learning with neural networks can give results similar to centralized methods without violating the principle of patient privacy. In more recent works, there was research using multimodal federated learning that uses MRI, cognitive tests, and biomarkers, along with using explainable AI to increase the reliability of results for clinicians (Mitrovska et al., 2024). Deep learning-centric, attention-based architectures, like Vision Transformers, hybrid CNN-Transformers, and attention-enhanced CNNs, provide a robust and flexible framework for processing different modalities in health care and global contextual relationships in medical images. Given their capability to model long-range dependencies, these architectures are tailored for use in multimodal health care. Nevertheless, their use is limited due to high computational complexity, large data requirements, and increased communication overhead, thus making these architectures sub-optimal for federated learning scenarios.

### 2.4 Summary of gaps

Based on the literature, three gaps can be identified:

- CNNs are strong image biomarkers that do not possess contextual reasoning.
- Deep multimodal models have infrequently been used together with Fuzzy inference to enhance interpretability.
- Federated multimodal systems are available, although none of them combine privacy, multimodal fusion, and explainability.

FuzzyFed-CNN contributes to the field by solving all three and providing a scalable, interpretable, and privacy-preserving model for early AD detection.

## 3 Methodology

The architecture of the FuzzyFed-CNN Multimodal System of Fuzzy consists of sequential processing stages. These stages encompass the processing of loading the dataset, preprocessing, feature extraction, processing the CNN branch, processing the FIS branch, multimodal fusion, physician/expert systems, classification, and output of the model.

**Figure 1:**
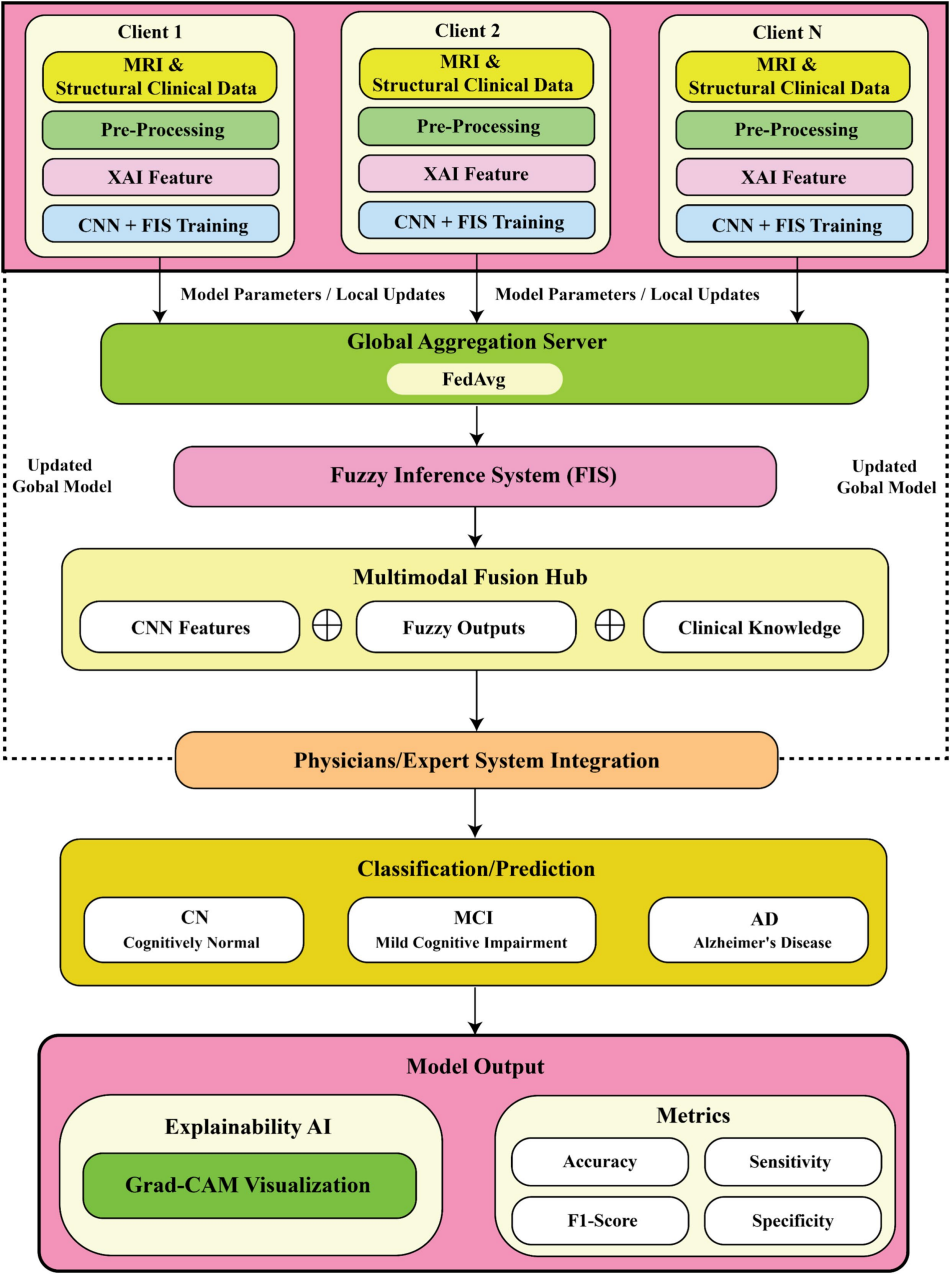
Overall architecture of FuzzyFed-CNN multimodal system (conceptual diagram – no experimental output).

As shown in Figure 1, the architecture of FuzzyFed-CNN employs FL and multimodal data integration to detect AD in its initial stage. The architecture takes three primary inputs: MRI neuroimaging data, derived structural biomarkers (e.g., hippocampal volume), and clinical metadata (age, MMSE score, etc.). The inputs were subjected to normalization, augmentation, and feature scaling so that there is uniformity amongst the data across the distributed client nodes. The pre-processed data is processed through two parallel branches: (1) the CNN branch, where the model runs several convolutional, pooling, and activation layers to extract the cortical thinning-hippocampal atrophy and other neuroanatomical biomarkers, and (2) the Fuzzy Inference System (FIS) branch, where fuzzy membership functions, rule-based logics, and the clinical data is used to model the uncertainty and linguistic variability of the data. The proposed framework utilized 2 stage fuzzy inference mechanisms. In the first FIS, feature level uncertainty modeling was done and within the second FIS decision level reasoning was done during multimodal fusion. It is crucial as it will improve interpretability and robustness. CNN plays a crucial role in this case as it effectively extracts special features with low computational complexity. The outputs of both branches are combined in the multimodal fusion layer to produce a hybrid feature vector, which contains the spatial data from the imaging and the clinical data. This representation helps to predict one of the following three cognitive states: Cognitively Normal (CN), Mild Cognitive Impairment (MCI), and AD. Consistent with principles of FL, the participating hospitals each retain full control of their data and do not share any raw data from their patients, ensuring legal compliance (HIPAA/GDPR). The local model parameters are sent to a global aggregation server, and through Federated Averaging (FedAvg), the local updates are synthesized into a single model, which is then sent back out to the participants for several more training rounds. Additional benchmark experiments were conducted with MobileNet, ResNet, DenseNet and EfficientNet. The findings suggest effectiveness and computational feasibility of the selected CNN backbone. Model interpretability is kept for Grad-CAM since it illustrates important portions of the image that drive the predictions for the most important image. Overall performance is measured using an integrated score that evaluates the accuracy, sensitivity, specificity, and F1 score. To ensure reproducibility following experimental settings were used in Table 1.

**Table 1:** Experimental setup settings.

Parameter category	Configuration/setting
Dataset sources	ADNI and OASIS multimodal Alzheimer’s datasets
Imaging modality	MRI neuroimaging data
Clinical features	Age, MMSE score, hippocampal volume, structural biomarkers
Data normalization	Min-Max normalization
Data augmentation	Rotation, flipping, zooming, intensity variation
Feature scaling	StandardScaler normalization
CNN backbone	Custom CNN architecture
Comparative models	MobileNet, ResNet50, DenseNet121, EfficientNet-B0
Input image size	224 × 224 pixels
Convolution kernel size	3 × 3
Activation function	ReLU
Pooling layer	Max Pooling (2 × 2)
Dropout rate	0.5
Batch size	32
Learning rate	0.001
Optimizer	Adam Optimizer
Loss function	Categorical Cross-Entropy
Number of epochs	100
Federated learning algorithm	Federated Averaging (FedAvg)
Number of federated clients	5 decentralized institutions
Communication Rounds	50 rounds
Local training epochs	5 epochs per client
Fuzzy membership functions	Triangular and Gaussian membership functions
Fuzzy rules	Mamdani-based rule inference
Fusion strategy	Multimodal feature-level fusion
Explainability method	Grad-CAM visualization
Evaluation metrics	Accuracy, Sensitivity, Specificity, Precision, Recall, F1-score
Statistical validation	95% Confidence Interval, *p*-value analysis, Cohen’s d effect size
Hardware environment	NVIDIA GPU-enabled training environment
Software frameworks	Python, TensorFlow, Keras, Scikit-learn
Privacy preservation	No raw patient data sharing between institutions
Classification categories	CN, MCI, AD
Validation strategy	Stratified train-test split with cross-validation

The framework is designed to preserve data privacy while maintaining diagnostic accuracy and interpretability across multiple healthcare institutions.

### 3.1 Dataset description

The two datasets used in the study were the Alzheimer’s Disease Neuroimaging Initiative (ADNI) and the Open Access Series of Imaging Studies (OASIS-3), which were both popular neuroimaging datasets for detecting AD. In the paper, T1-weighted structural MRI scans were the sole imaging modality for feature extraction by CNN. The OASIS dataset link is https://www.kaggle.com/datasets/ninadaithal/imagesoasis.

Even though OASIS-3 has CT imaging data and ADNI has PET imaging data, these modalities were not added to the existing implementation to ensure consistency and to minimize computational complexity. Rather, the model concentrates on the data obtained through MRI along with systematized clinical variables, including age, MMSE scores, gender, and hippocampal volume. The ADNI sorted dataset link is https://www.kaggle.com/datasets/summaiyamahmood/adni-sorted-data.

MRI scans were used to measure the hippocampal volume, which has been determined to be an important input in the fuzzy inference system through automated segmentation. This ensures consistency between imaging features and clinical reasoning in the multimodal framework. Besides the neuroimaging data, demographic and neuropsychological variables were also included in the multimodal framework. These variables were age, gender, Mini-Mental State Examination (MMSE) scores, and hippocampal volume. The volumes of the hippocampus were obtained in MRI scans through an automated segmentation algorithm, and the mean value of the left and right hippocampus formed the input in the fuzzy inference system. These measures were done to avoid data leakage; hence, the partitioning of the datasets was conducted at the subject level instead of the image level. Each MRI scan of the same subject got into the same data partition (Paul-Chima Ugwu and Ogenyi, 2026). Only the training dataset was rotated and flipped as data augmentation methods to enhance the prediction of the model without leaking information. The implementation has focused on the MRI and the structured clinical features. The CT and PET modalities will be added in the future to enrich the multimodal capabilities.

This has been updated to enhance reproducibility and transparency in the data set description. In the manuscript, we have added detailed descriptions of the cohorts in terms of the total number of subjects, their classification (CN, MCI, and AD), demographics, and selection criteria. Also, we have elaborated on the partitioning process in which the training (70%), validation (15%), and testing (15%) subjects were allocated without overlap. Moreover, the details about the distribution of subjects among the five federated sites have been provided in Table 2. In case of missing values, median/mode imputation has been performed.

**Table 2:** Cohort characteristics, dataset allocation, and federated client distribution.

Parameter	Value
Dataset source	ADNI
Total subjects	403
Cognitively normal (CN)	187
Mild cognitive impairment (MCI)	87
Alzheimer’s disease (AD)	129
Training set (70%)	282
Validation set (15%)	60
Testing set (15%)	61
Federated client 1	81
Federated client 2	81
Federated client 3	81
Federated client 4	80
Federated client 5	80
Missing value handling	Median imputation for numerical variables; mode imputation for categorical variables
Inclusion criteria	Subjects with valid MRI scans and corresponding diagnostic labels
Exclusion criteria	Subjects with corrupted MRI scans, incomplete imaging records, or missing diagnostic labels
Data partition strategy	Subject-level split performed before MRI slice extraction
Information leakage prevention	No subject overlap between training, validation, testing, or federated clients

#### 3.1.1 Data leakage prevention strategy

In order to avoid information leakage and an inflated estimate of the classification performance, prior to slice extraction from MRI volumes, the samples were grouped based on subjects at the level of partitions. Each MRI scan and its resulting axial slice corresponding to the same subject were grouped together in the same partition. Hence, there was no overlap among the subjects used for training, validation, and test datasets. The repeated scans of the same subject were also placed in the same partition, and no splitting occurred among different subsets (Yu et al., 2026). After partitioning, MRI volumes were sliced, and the prediction results of the slices per subject were aggregated by majority voting. In the Federated Learning setting, each subject belonged to only one federated client.

### 3.2 Local client data: privacy-preserving multimodal inputs

Local clients in the FuzzyFed-CNN configuration are separate hospitals, diagnostic facilities, or research centres with patient data. Due to strict healthcare regulations like HIPAA and GDPR, data is never shared among these institutions directly. Training happens locally, and only model parameters or gradients are transmitted to a central server, where they are aggregated.

Each client maintains two key data modalities:

1. MRI neuroimaging data
2. Structured clinical data (numerical and categorical)

**Figure 2:**
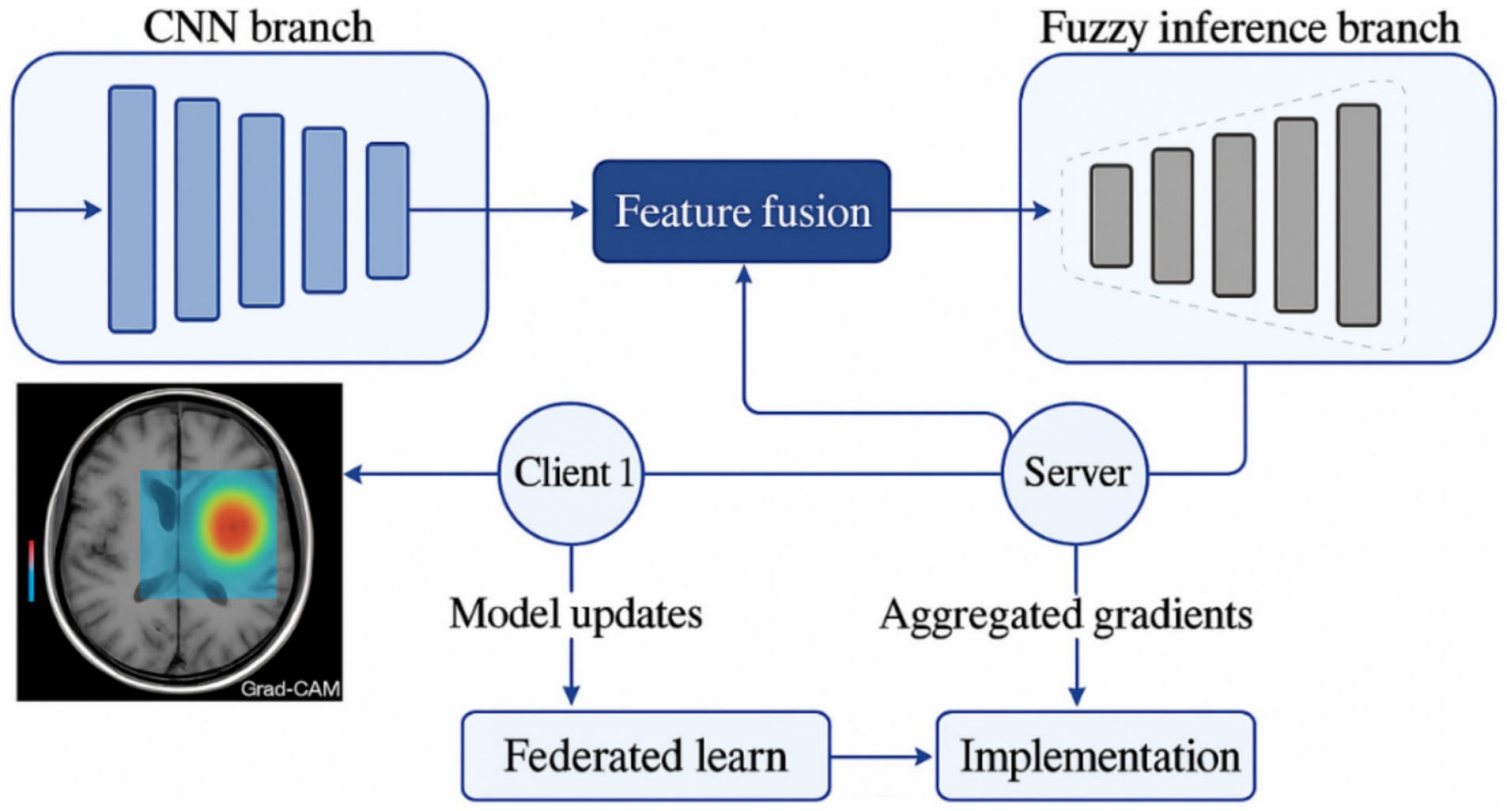
FL architecture with CNN–fuzzy inference dual branches and Grad-CAM-on brain MRI (conceptual diagram – no experimental output).

The multimodal input required for the proposed model is presented in Figure 2, representing MRI input, which serves as a primary modality used for feature extraction and analysis.

#### 3.2.1 MRI neuroimaging data (image modality)

MRI scans are rich in spatial and anatomical information. Each image is treated as a high-dimensional input tensor:

$$X_{\mathrm{MRI}}\in R^{H\times W\times C}$$ (1)

where:

- H: Height of the image (e.g., 128 px)
- W: Width of the image (e.g., 128 px)
- C: Number of channels (usually 1 for grayscale, 3 for RGB)

These MRI images are passed through a Convolutional Neural Network (CNN) to extract deep spatial features:

$$\;f_{\mathrm{img}}=\mathrm{CNN}\;(X_{\mathrm{MRI}})$$ (2)

The CNN may consist of several convolutional, ReLU, pooling, and dense layers. The output $f_{\mathrm{img}}$ is a feature vector representing image-based patterns such as brain tissue atrophy or structural anomalies linked to AD.

#### 3.2.2 CNN backbone selection justification

Though the data in MRI is three-dimensional in nature, this experiment used a two-dimensional CNN network on axial slices of MRI. The computational complexity can be greatly minimized with the help of the 2D CNN and can be easily trained in a FL environment where the computational resources can differ across institutions. The values in each MRI volume were broken down to axial slices, and slice-level predictions were aggregated through majority voting to give one patient-level classification. CNN was selected as a primary backbone model as it exhibits strong capability in automatic feature extraction, computational efficiency and it is suitable for medical image analysis. CNN is effective in learning hierarchical special representation from emerging data. One of the major reasons of selecting CNN is its ability to automatically extract discriminative local and global features. It extracts features without requiring manual feature engineering. From a practical perspective, CNN offers lower competition and complexity. As the proposed framework combines Federated learning, computational efficiency becomes an important consideration. Lightweight CNN architecture reduces the communication cost and memory consumption as well. Thus, it becomes feasible to use it in distributed Federated environments (Poulakis and Ioannou, 2026). There also exist architectures including ResNet, DenseNet and EfficientNet which have excellent performance in several computer vision tasks. These models are typically deeper and computationally more complex. The transformer-based architecture achieves strong global feature modeling they require large scale datasets and extensive computational resources. When compared to these models, CNN backbone provides a balanced tradeoff between diagnostic performance and computational efficiency. CNN Backbone also offers training stability and Federated deployment feasibility. Whereas recent models like Vision Transformers (ViTs), Swin Transformers, DenseNet, EfficientNet, and ResNet have shown their effectiveness in medical image analysis, they need a large number of data points during the training phase and higher computational requirements as well. On the other hand, CNN models represent a better trade-off between the ability to extract features and ease of implementation. In order to further investigate the effectiveness of CNN models, experiments were also performed using MobileNet, ResNet50, DenseNet121, and EfficientNet-B0 under similar training conditions. As will be seen from Section 4, the FuzzyFed-CNN model has demonstrated better performance in this experiment as well.

#### 3.2.3 Clinical data (tabular modality)

Structured clinical data includes:

- Numerical features: age, MMSE score, hippocampus volume
- Categorical features: gender, education level, genetic markers (e.g., APOE4)

Let the structured input vector be:

$$X_{\text{clin}}=[\;x_{1},,x_{2},,.\ldots ..x_{n}]\in R^{n}$$ (3)

These features are passed through a FIS to model uncertainty and linguistic rules (e.g., “IF age is high AND MMSE is low THEN risk is high”).

$$\;\;\;\;f_{\text{fuzzy}}=\mathrm{FIS}\;(X_{\text{clin}})$$ (4)

Where $f_{\text{fuzzy}}$ captures symbolic or rule-based reasoning over patient metadata.

Both $f_{\mathrm{img}}$ , $f_{\text{fuzzy}}$ vectors are forwarded to the Multimodal Fusion Layer

$$f_{\text{fused}}=\text{Concat}\;(f_{\mathrm{img}},f_{\text{fuzzy}})$$ (5)

This fused vector is then used locally for model training before being aggregated federatively. The complete FuzzyFed-CNN training procedure is summarized in Algorithm 1.

ALGORITHM 1 FuzzyFed-CNN training with FedAvg - including client selection, local epochs, and weighted aggregation.
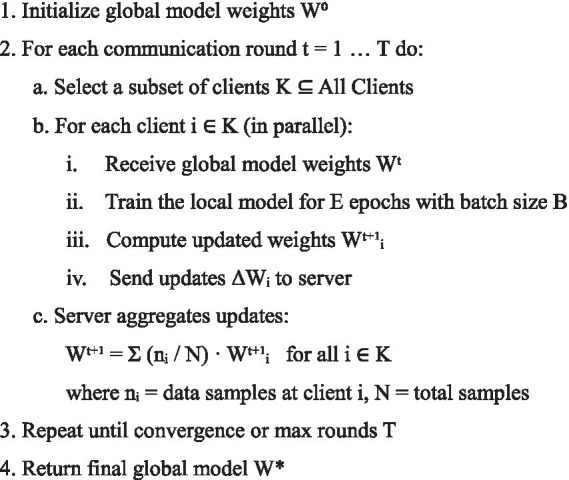

On each local client in the FuzzyFed-CNN platform, two complementary modalities are processed. Structural information about the brain at high dimensionality is obtained through the MRI neuroimaging data, so the CNN can learn spatially complex patterns associated with large-scale neurodegeneration, like cortical thinning or ventricular enlargement. This yields the feature vector f_img with fine-grained visual features (Fan et al., 2026). While that occurs, the systematic clinical data in the form of numerical (e.g., age, MMSE scores) and categorical (e.g., gender, APOE4 status) variables are run through a FIS to generate f_fuzzy, simulating expert-level decision rules under uncertainty. Integrating these fuses’ objective image information and subjective clinical judgment to generate a solid, multimodal diagnosis.

The architecture depicts a multimodal FL system in which MRI scans and structured clinical data are processed by two parallelized streams: a CNN stream that learns spatial neuroanatomical features related to the pathology of Alzheimer’s, and a fuzzy inference stream, which learns clinical reasoning based upon semantic interpretation performed with rules. Both branches have their outputs selectively fused into a single feature fusion layer, to create a unit multimodal representation that is finally learned locally at each of the participating clients (e.g., a hospital node) without exchanging raw data. The updates made to the model are sent in a secure way to a central federated server, where gradient aggregation is carried out in FedAvg to create a global model generalizing across institutions without violation of HIPAA/GDPR-compliant privacy. Following international revisions, explainability systems (e.g., Grad-CAM) can identify clinically significant regions (e.g., hippocampal atrophy) to enhance medical interpretation and enhance confidence in the diagnostic output.

### 3.3 Dual-modal feature extraction

Within the FuzzyFed-CNN design, the diagnostic strength is attained through the manipulation of two complementary modalities of data: neuroimaging and clinical metadata. Both modalities add a separate dimension of information that is vital in effective and interpretable diagnosis of the disease.

#### 3.3.1 CNN model (image modality)

MRI neuroimages are analyzed with CNNs and used to identify fine spatial and anatomical features suggesting neurodegenerative disease, including cortical thinning or hippocampal shrinkage. The raw image is represented as a 3D input-tensor first. As per Equation 1.

Where:

- H: Height of the image
- W: Width of the image
- C: Number of image channels (typically 1 for grayscale MRI)

The CNN architecture processes this tensor through a sequence of convolutional, activation, and pooling layers to output a deep visual representation, as per Equation 2. Here, $f_{\mathrm{img}}\in R^{d}$ is a compact, high-dimensional vector encoding critical spatial biomarkers.

#### 3.3.2 FIS for tabular data modality

Membership functions corresponding to input variables, and clinical metadata specified within Table 3, comprising both numerical and categorical features, contain patient information such as age, MMSE score, education level, and genetic risk factors (e.g., APOE4 allele). These features are first assembled into a structured vector, as per Equation 3. Because clinical data often involves ambiguity and imprecision, a FIS is applied to encode rule-based reasoning, as per Equation 4. Where $f_{\text{fuzzy}}=\in R^{m}$ is a symbolic feature vector derived from expert-defined fuzzy rules (e.g., “IF age is high AND MMSE is low THEN risk is high”). To combine uncertainty and expertise in the diagnosis process, the FIS has been devised. The input variables, which were age, MMSE score, and hippocampal volume, were retrieved through mapping to fuzzy membership functions that were linguistic expressions such as low, medium, and high. To take an example, age was broken down into fuzzy sets {young: <55, middle-aged: 55–65, elderly: >65} whereas MMSE was split into sets {normal: 27+, slight decline: 24–26, poor: <24}. Based on such membership functions, it was possible to formulate rules to represent clinical reasoning. The following is illustrated by a typical example: IF age > 65 AND MMSE < 24 THEN risk = HIGH. Mamdani-type reasoning was utilized to perform the entire process of inference, and centroid defuzzification brought about the resulting outputs. This enabled such a model to use symbolic, human-like decision rules in addition to numerical MRI features, making them more interpretable.

**Table 3:** Membership functions associated with input variables.

Variable	Fuzzy set	Range/definition
Age (years)	Young	< 55
	Middle-aged	55–65
	Elderly	> 65
MMSE score	Normal	≥ 27
	Slight decline	24–26
	Poor	< 24
Hippocampal volume	Normal	≥ mean + SD (dataset-specific)
	Mild atrophy	mean ± SD
	Severe atrophy	≤ mean – SD
Risk (Output)	Low	Defuzzified score: near 0
	Medium	Defuzzified score: mid-range
	High	Defuzzified score: near 1

Table 4 provides the fuzzy rule base of Alzheimer prediction with Fuzzy-CNN. Age, MMSE score, and hippocampal volume are some clinical features incorporated in the fuzzy rule base as symbolic reasoning to assess the risk, and each rule represents a clinically significant situation. An example is a High-Risk rule, which is the combination of several negative factors, e.g., advanced age, bad MMSE performance, and intense hippocampal atrophy (Rule 1) or when severe atrophy only is present, irrespective of other inputs (Rule 10), because the structural brain degeneration is a potent biomarker. Medium Risk is often attributed when there is a presence of one or two risk factors, but not all, like patients with old age and normal MMSE (Rule 3) or Preservation in the middle-aged with slight deterioration and mild atrophy (Rule 5). Low Risk occurs when protective factors are predominant, e.g., middle-aged or young persons with normal scores on cognitive abilities and intact brain volume (Rules 6 and 8). The advantage of this is that this model picks up both additive and dominant effects: additive because a combination of moderate risk factors results in medium risk, and dominant because one strong risk factor, such as severe atrophy, can dominate others. Therefore, the rules replicate clinical reasoning, trade uncertainty, and clarify the system by coding human-like decision-making behaviors into a structured and interpretable system.

**Table 4:** Fuzzy rule base for Alzheimer’s prediction.

Rule no.	Age	MMSE	Hippocampal volume	Risk
1	Elderly	poor	Severe atrophy	High
2	Elderly	Slight Decline	Mild atrophy	Medium
3	Elderly	Normal	Normal	Medium
4	Middle-aged	Poor	Severe atrophy	High
5	Middle-aged	Slight decline	Mild atrophy	Medium
6	Middle-aged	Normal	Normal	Low
7	Young	Poor	Severe atrophy	Medium
8	Young	Normal	Normal	Low
9	Elderly	Poor	Normal	Medium
10	Any	Any	Severe atrophy	High

Fuzzy membership functions and rule base construction was based on clinical parameters which can be considered as signs of development of the disease, such as age, MMSE rating, and hippocampal volume. Linguistic values were chosen considering generally accepted criteria of neuropsychological tests and imaging. In particular, MMSE values less than 24 are usually regarded as evidence of cognitive impairment, whereas hippocampal atrophy serves as an important biomarker of the disease under discussion. In order to test the robustness of the presented fuzzy reasoning mechanism, its sensitivity to changes of boundary values of membership functions in a predetermined range around each threshold was analyzed. As a result, the classification accuracy did not change significantly, suggesting that the constructed model did not rely strongly on any specific threshold value (Soliman et al., 2026). Hence, this approach can be viewed as clinically meaningful and reliable enough for practical applications.

#### 3.3.3 Fusion and complementarity

**Figure 3:**
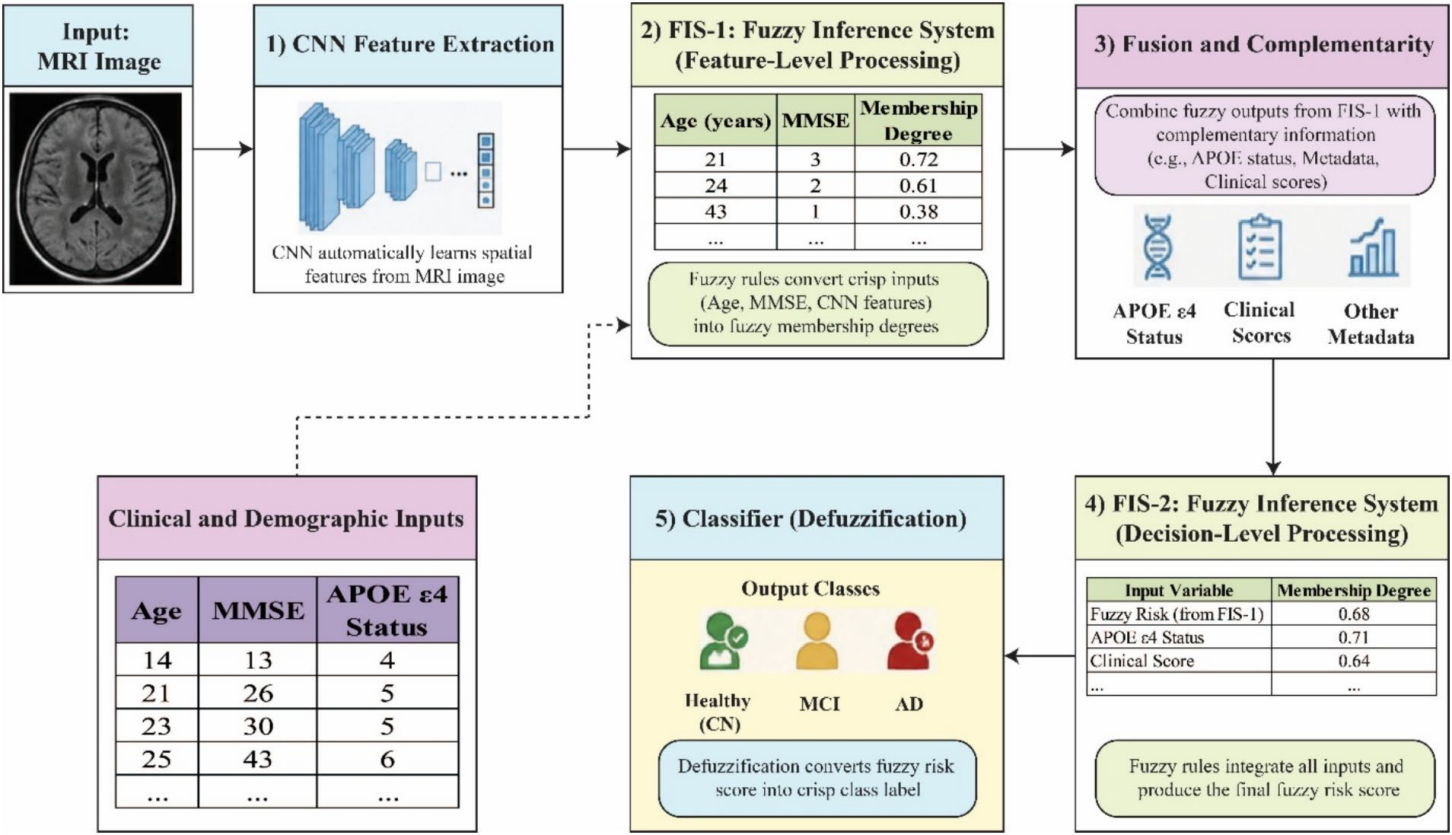
CNN–FIS fusion framework for Alzheimer’s prediction (conceptual diagram – no experimental output).

The feature extraction with the use of CNN architecture is given in Figure 3. The vectors from the CNN and FIS branches are then fused, as per Equation 5. This fusion combines spatially derived imaging biomarkers with semantically rich clinical insights, enabling the model to reason both *numerically* and *linguistically*. The multimodal feature vector $f_{\text{fused}}\in R^{d+m}\;$ serves as the input to subsequent classification layers during training and federated aggregation. This dual-path architecture thus ensures both deep pattern recognition from imaging and explainable clinical reasoning, significantly improving diagnostic reliability and transparency.

In order to make the process more transparent, the fuzzy inference engine is employed in two stages of the processing workflow. First, the FIS will conduct the uncertainty modeling of the features through converting the clinical variables in numerical values into linguistic ones. The next FIS is the one that makes decisions using the results of multimodal fusion as well as the CNN features and fuzzy clinical data. The dual-modal feature extraction from FuzzyFed-CNN enables diagnostic precision with the extraction of visual biomarkers and semantic clinical patterns. The CNN module identifies anatomical lesions in MRI scans, such as cortical atrophy, while the FIS handles fuzzy or imprecise clinical inputs via expert-specified rules. The dual-modal complementarity, merging data-driven learning and symbolic reasoning, allows the system to conduct explainable and strong diagnoses. By combining structural image features with tabular risk factors, the model is able to identify early symptoms of AD more accurately, yet at the same time ensure interpretability essential in clinical trust and real-world healthcare decision-making settings.

### 3.4 Multimodal fusion

In the FuzzyFed-CNN framework, multimodal fusion plays a critical role by uniting two distinct feature vectors: image-derived features f_img from the CNN and symbolic clinical features f_fuzzy from the FIS. Mathematically, this integration can be written as: As per Equation 5. This hybridized vector $f_{\text{fused}}\in R^{d+m}$ now reflects an overall picture of the state of the patient, including both spatial pathology and semantic clinical risk factors. This means that the downstream classifier can use the qualities of this hybrid to better generalize and make decisions. The combination also makes sure that the model is not based on anatomical knowledge (e.g., brain atrophy) or symbolic explanations (e.g., rule-based risk), but rather a combination of both viewpoints to generate more reliable and interpretive diagnostic forecasts. The multimodal fusion approach enhances the capacity of the model to identify correlations of physical degeneration (e.g., MRI patterns) with abstract health outcomes (e.g., MMSE scores). It enhances strength and clinical significance, particularly in sophisticated neurological disabilities such as Alzheimer.

### 3.5 Federated learning framework

FuzzyFed-CNN leverages FL to facilitate privacy-respecting, distributed model training across the clinical institutions. Instead of transferring raw patient data between institutions, FL allows each participating site to train models locally while sharing only model parameters with a central aggregation server. Although regulated data sharing may be possible under privacy frameworks such as GDPR and HIPAA when appropriate safeguards are applied, FL provides an additional privacy-preserving mechanism that reduces the need for direct data exchange while enabling collaborative model development. This collective process is regulated by the FedAvg algorithm. Even though sharing medical data may be provided under controlled conditions based on pseudonymized datasets, FL is also proposed, which offers another approach according to which model training can be provided collaboratively without moving raw patient data across institutions. The local model weights at each client are updated using gradient descent on the local loss, as defined in Equation 6.

The process can be summarized mathematically:

1. Local model update:

$$\;w_{i}^{\left(t+1\right)}=w_{i}^{\left(t\right)}-n\nabla \tau_{i}(w_{i}^{\left(t\right)})$$ (6)

where:

-
$w_{i}^{\left(t\right)}$ : model weights of client i at round t
-
$\tau_{i}$ : local loss function
-
n : learning rate

1. Federated Aggregation (FedAvg):

$$w^{\left(t+1\right)}=\sum_{i=1}^{k}\frac{n_{i}}{n}\;w_{i}^{\left(t+1\right)}\;\;\;$$ (7)

where:

-
$w^{\left(t+1\right)}$ : global model weights
-
$n_{i}$ : number of samples on client i
-
n : total samples across all clients
- K: number of clients

This approach guarantees each client contributes proportionally to the global model as a function of the size of its data, maintaining fairness and enhancing generalization. FL enables safe, collaborative training without the need to store data centrally. It guards sensitive patient records while still taking advantage of distributed data diversity, which is critical to developing strong, real-world diagnostic models across institutions.

In FuzzyFed-CNN, a FL framework is used to maintain privacy in the institutions where the training is to be done. At the initialization stage, the worldwide model is initiated and reproduced with chosen clients, such as hospitals or diagnostic facilities. All clients utilize their own multimodal data in training the model (e.g., the features of the MRI diagnosis provided by CNNs and clinical features computed through fuzzy inference). Local training is sent to the central server, but only the newly obtained model parameters, which will then be combined with the FedAvg algorithm. Then this process is repeated a few rounds of communications till the model converges. In order to manage non-IID data across clients, weighted averaging and normalization are used so that contributions are fair. Minimization of communication expenses is achieved by limiting the amount of data to be transferred and having efficient sharing of parameters. Convergence analysis indicated that the framework was efficient and scalable and converged around 100 communication rounds.

#### 3.5.1 Justification for FedAvg

The aggregation strategy has been selected as FedAvg for three reasons:

- Established stability: FedAvg has been extensively tested in studies of multimodal FL in terms of its ability to work with heterogeneous clients.
- Computational efficiency: FedAvg has less communication and computation overhead compared to more sophisticated methods (FedProx, FedDyn), an important factor when operating in multi-institutional systems.
- Extension baseline: Although FedProx has shown an enhancement over statistical heterogeneity, initially, we sought to achieve a good baseline; we point to Future Work to deal with non-IID distributions.

This trade-off guarantees scalability and reproducibility whilst being fair amongst customers.

A federated learning framework involving five healthcare institutions in a simulation setup was utilized. Equal distribution of subjects was ensured among the clients without any overlapping between institutions in terms of subject isolation. A nearly Independent and Identically Distributed (IID) distribution approach was chosen to ensure balanced diagnosis among clients. All five clients took part in performing local training and updating model parameters at each communication round. Aggregation through FedAvg algorithm was done taking into account the weighting according to the sample size of each client. Training of five local epochs was achieved by each client in each round utilizing a batch size of 32 and Adam optimizer. Training continued until 50 rounds while convergence was determined by checking the stabilization of the validation loss. Model was deemed to have converged when there is no more improvements observed in the validation loss. Experiments were carried out on a machine equipped with an NVIDIA GPU with TensorFlow and Keras packages. Federated aggregation involved only model parameters while patient records are kept locally for privacy purposes. While the proposed framework was shown to exhibit satisfactory convergence and scalability, direct measurement of its communication bandwidth utilization, network latency, and communication overhead were not considered within the scope of this current work. Further research will address such practical considerations within large-scale implementation settings using real-world healthcare facilities with more participant organizations involved.

### 3.6 Global diagnostic model

Following each communication round, client updates are aggregated via FedAvg to form the global model, which is broadcast back to clients. Iteration over rounds yields a model that generalizes across site-specific distributions and both modalities, improving robustness without exchanging raw data. The global model update after each communication round is given by: As per Equation 7. This updated global model $w^{\left(t+1\right)}$ is then redistributed to all participating clients for the next local training cycle, allowing continual improvement while preserving data locality. The worldwide model models generalized diagnostic patterns that arise from heterogeneous patient populations and multimodal inputs from institutions. Such heterogeneity contributes to robust model performance, less data bias overfitting, and supports clinically sound decision-making within a greater real-world setting. The strategy, therefore, harmonizes privacy, performance, and scalability essential in contemporary medical AI use. The MRI features were extracted by means of the CNN architecture that provided four convolutional layers with a 3×3 filter size, an activation layer with ReLU activation, and dimension reduction activation using a max pooling layer. A normalization function (batch normalization) was used after every convolution layer in order to enhance the stability, and dropout layers (*p* = 0.4) were added to avoid learning rather than generalization. The feature maps that were extracted were flattened and underwent two fully connected layers with sizes 128 and 64, after which they were fused with fuzzy clinical features at the multimodal fusion layer. The Adam optimizer was employed in training with a learning rate of 0.0001 and at a batch size of 32. The federated framework trains the model over 50 rounds of communication, during which local training is used 5 epochs by each client during each communication round. Since there was a risk of overfitting, early stopping was used, with the loss on validation used to check it. The experiments were carried out the data were separated into training (70%), validation (15%), and testing (Bernecker et al., 2022) sets. The use of an NVIDIA Tesla V100 GPU server, using 32 GB of memory, could efficiently train the multimodal dataset.

### 3.7 Explainability with Grad-CAM

Grad-CAM determines the significance of every feature map in the final convolutional layer using the gradients of the class score prediction with respect to the feature maps, as given in Equation 8.

$$\alpha_{k}^{c}=\frac{1}{Z}\;\sum_{i}\sum_{j}\frac{\delta y^{c}}{\delta A_{\mathrm{ij}}^{k}}$$ (8)

Where:

-
$\alpha_{k}^{c}$ is the importance weight for feature map k,
-
$y^{c}\;$ is the class score for the predicted class c,
-
$A_{\mathrm{ij}}^{k}$ is the activation at spatial location (i,j) in feature map kkk,
- Z is the total number of pixels in the feature map.

#### 3.7.1 The Grad-CAM heatmap

The final class-discriminative localization map is then obtained by a weighted combination of the feature maps followed by a ReLU, as expressed in Equation 9.

$$L_{\text{Grad}-\mathrm{CAM}}^{c}=\text{ReLU}\;(\sum_{k}\alpha_{k}^{c}\;A^{k})$$ (9)

**Figure 4:**
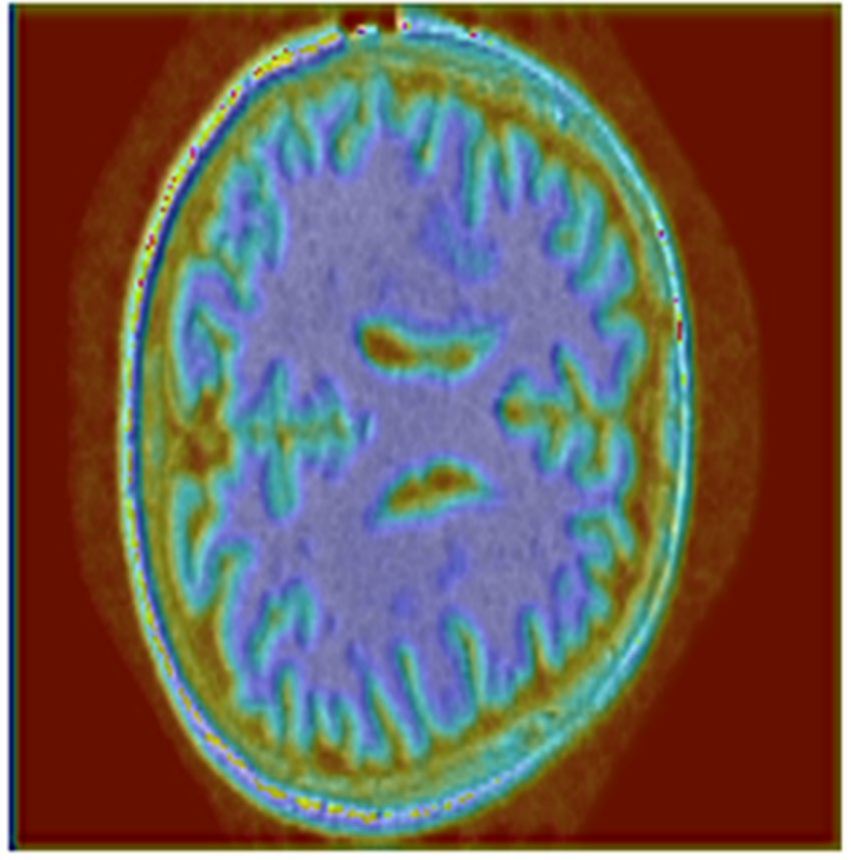
Grad-CAM heatmaps overlaid on MRI slices, highlighting hippocampal and cortical activation regions.

This heatmap Figure 4 is subsequently overlaid onto the initial MRI image, spatially identifying the areas (e.g., hippocampus, cortex) that resulted in the model labeling a patient as at-risk for AD.

#### 3.7.2 Clinical interpretability

The hippocampus and cortical regions of the brain, which are emphasized by the Grad-CAM visualizations, are recognized biomarkers of the pathology of AD. There is a strong correlation between cortical thinning and global neurodegenerative change and hippocampal atrophy, and memory loss. Hippocampal atrophy correlates strongly with memory decline and cortical thinning with global neurodegenerative change. The convergence of model attention to these clinically validated structures adds to interpretability and further gives confidence that FuzzyFed-CNN makes its predictions on meaningful neuroanatomical features instead of spurious correlations.

**Figure 5:**
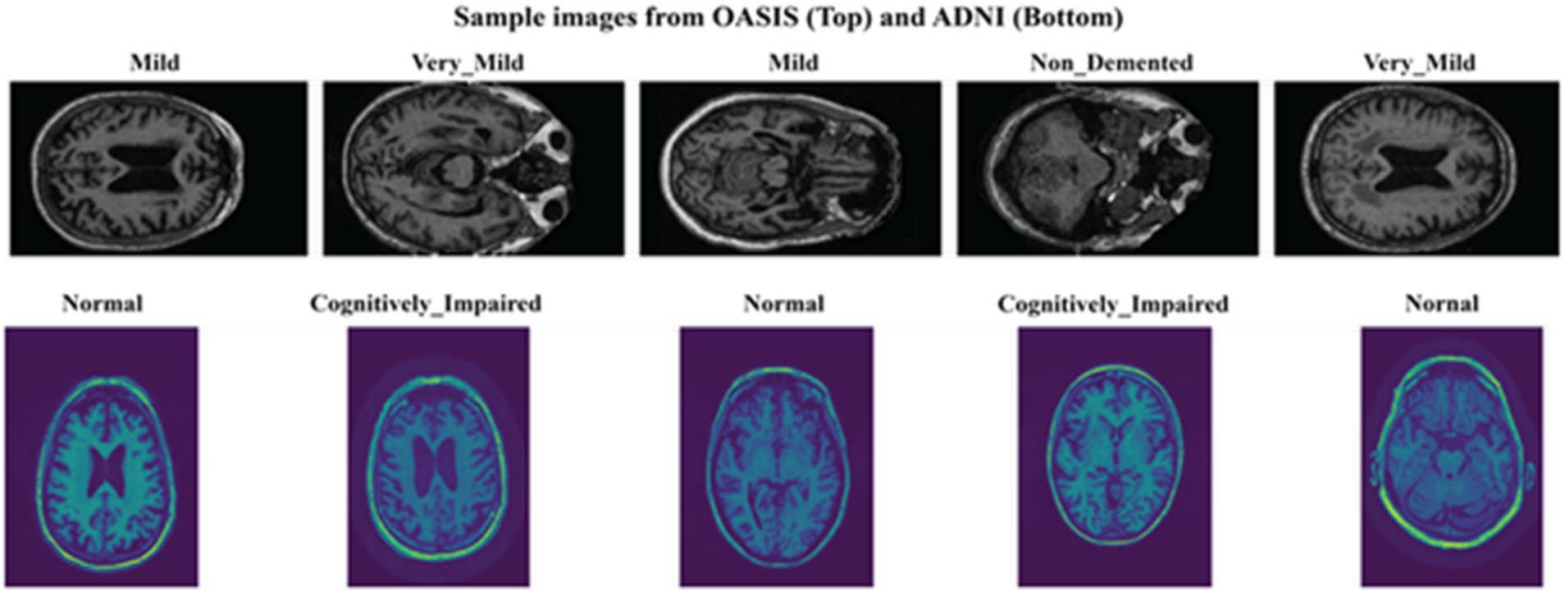
Sample MRI images from OASIS (top row) and ADNI (bottom row) datasets showing different dementia stages.

**Figure 6:**
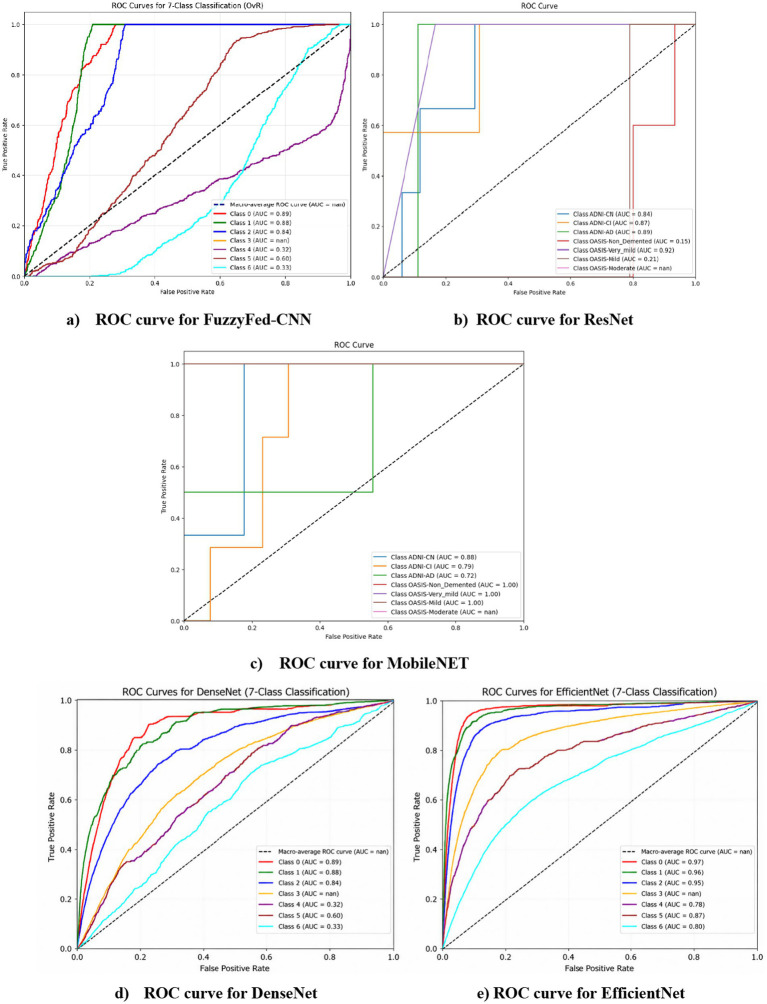
ROC curves comparing FuzzyFed-CNN, MobileNet, ResNet, DenseNet and EfficientNet (AUC shown). **(a)** ROC curve for FuzzyFed-CNN. **(b)** ROC curve for ResNet. **(c)** ROC curve for MobileNET. **(d)** ROC curve for DenseNet. **(e)** ROC curve for EfficientNet.

**Figure 7:**
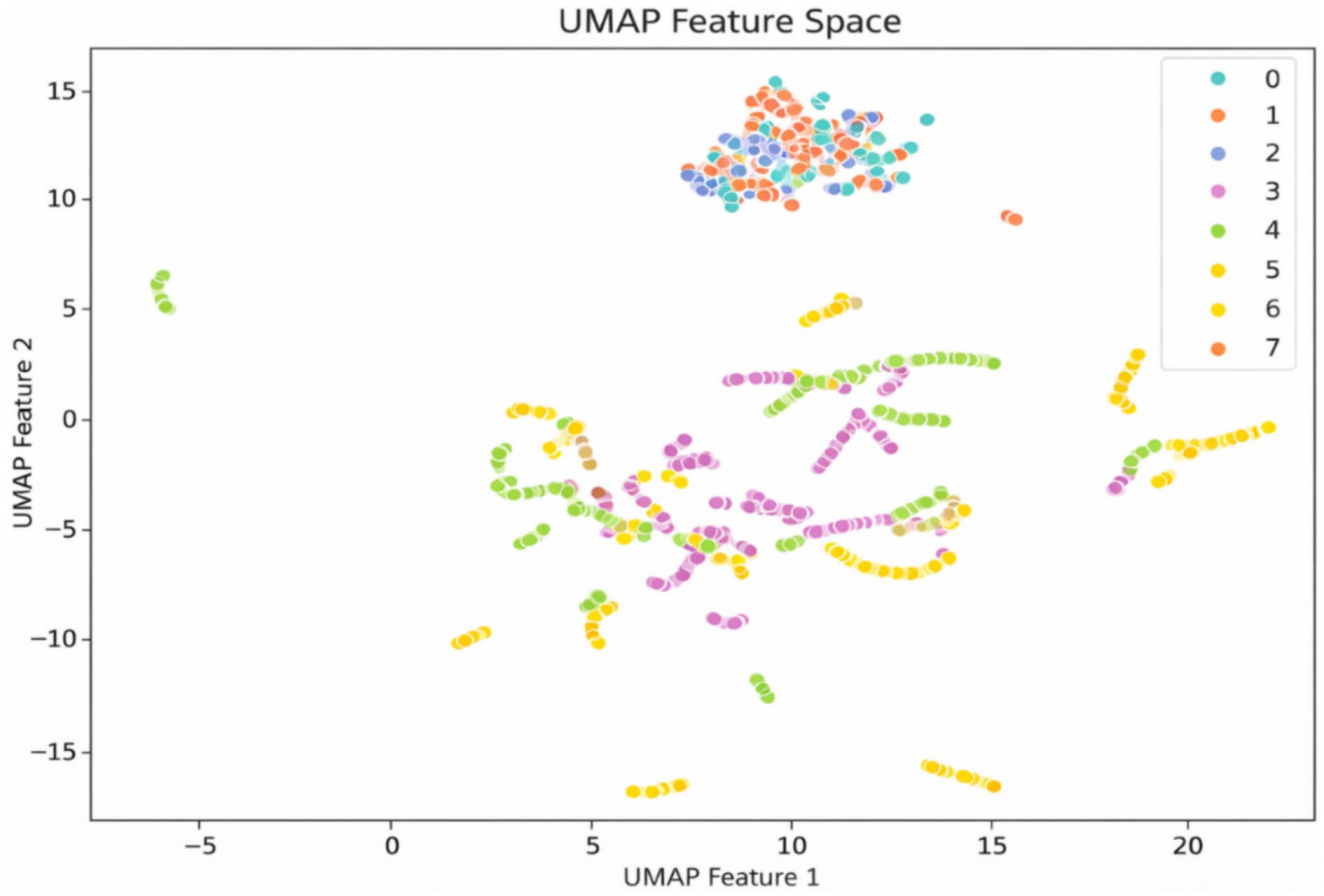
UMAP projection of learned multimodal features showing group separation.

**Figure 8:**
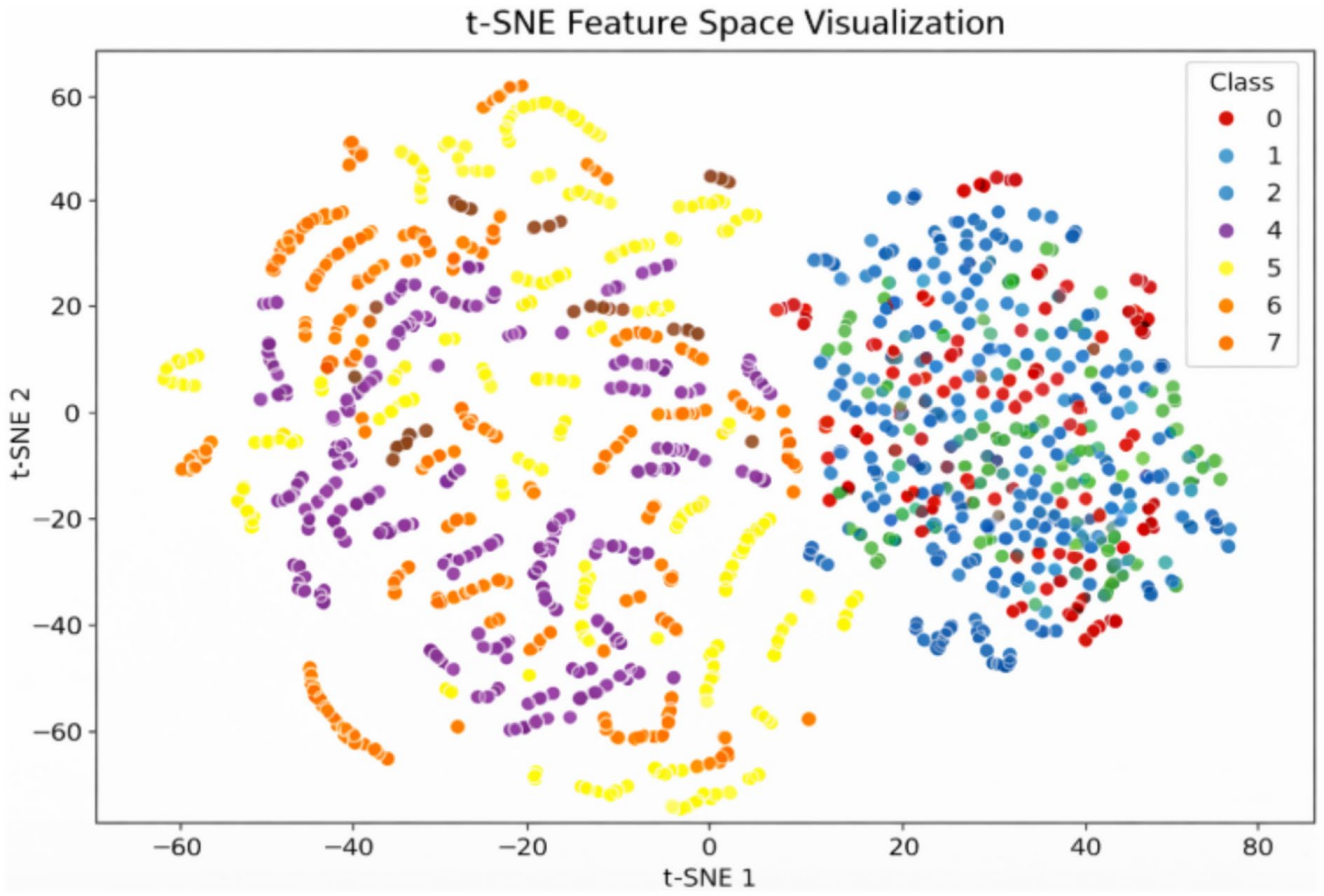
t-SNE projection of learned multimodal features.

**Figure 9:**
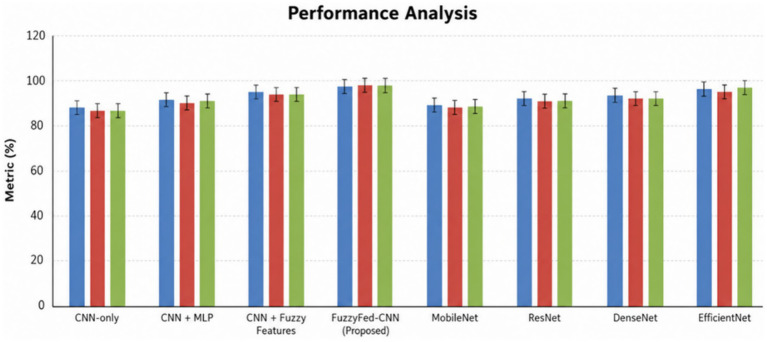
Performance comparison with different models and metrics.

**Figure 10:**
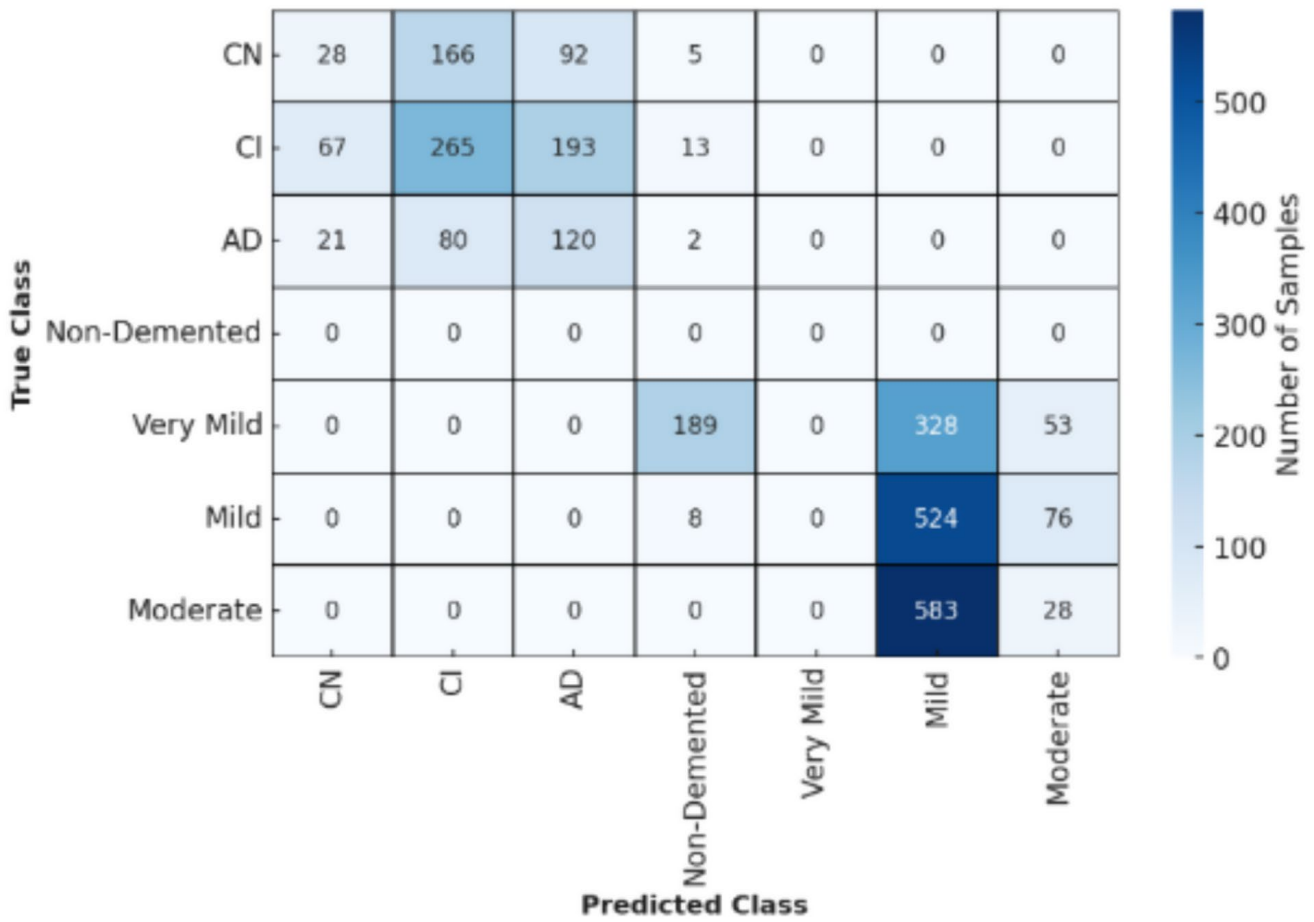
Confusion matrix heatmap of the FuzzyFed-CNN on ADNI and OASIS datasets (macro averaged over five-fold cross-validation).

**Figure 11:**
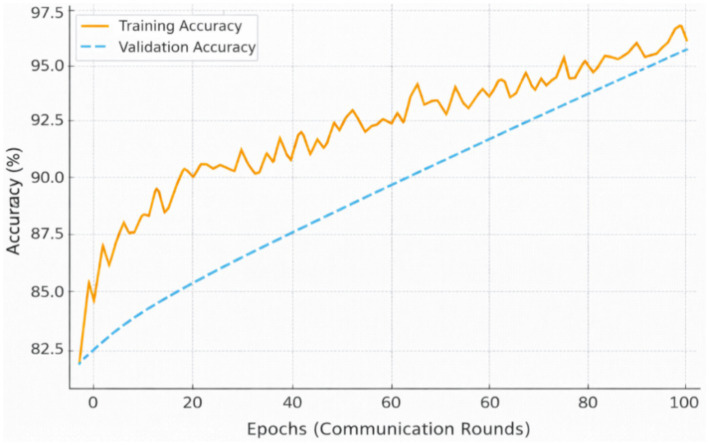
Training and validation accuracy epochs.

**Figure 12:**
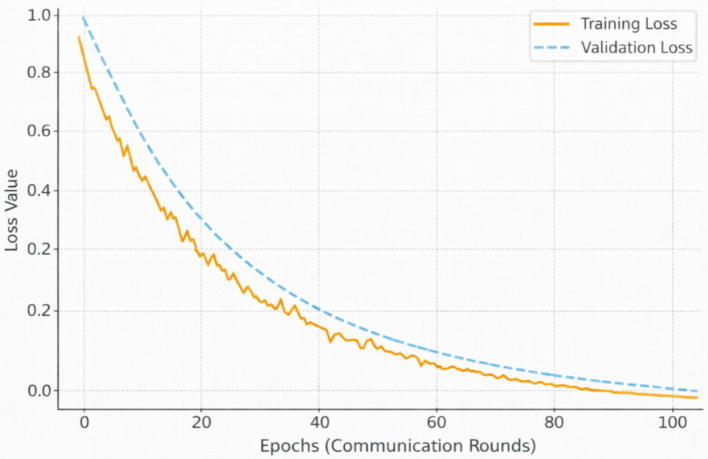
Eighty epochs of training and validation loss.

**Figure 13:**
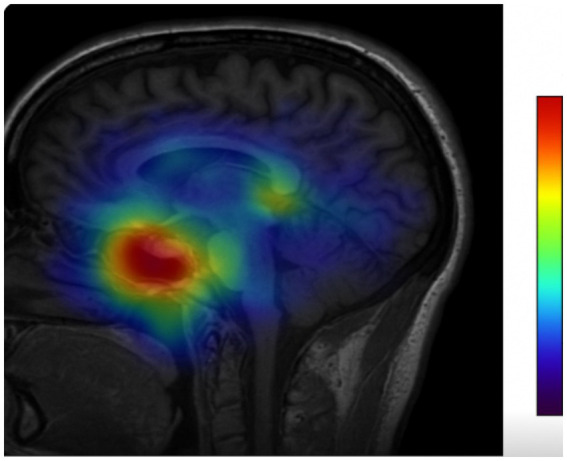
Grad-CAM visualization.

Even though Grad-CAM maps showed that the trained neural network reliably localized clinically important neuroanatomy such as the hippocampus and cortex, the current paper applied explainability via qualitative assessments mostly. Using quantitative measures of explainability based on annotation by expert neuroradiologists or using metrics like localization accuracy or ROI-based overlap analysis was outside the current scope. However, despite the lack of quantitative assessment, the resulting activation maps showed alignment with existing literature on biomarkers associated with Alzheimer’s disease. Further developments will include quantitative methods of assessing explainability guided by experts. In order to make things clearer, the figures used in this paper have been grouped into two groups. Figures 1–3 contain the diagrams showing the architecture, multimodal fusion technique, fuzzy inference process, and federated learning method of the suggested FuzzyFed-CNN model. On the other hand, Figures 4–13 show the outputs produced through experimentation within this study such as Grad-CAM maps, ROC curves, and projections in the feature space, confusion matrix, comparative analyses, and statistical results.

## 4 Results

All figures were redrawn by enabling high-quality rendering for publication purposes. The new figures make the visualization of the model structure, feature fusion steps, explanation outputs, and evaluation results more comprehensible. Given that the classification problem parameters are three diagnostic groups (CN, MCI, and AD), sensitivity and specificity were determined with the one-vs-rest approach per group. The reported values are macro averages value in all the classes where there is equal consideration of diagnostic groups. The accuracy, sensitivity, specificity, and F1-score were evaluated for performance, as well as precision, recall, and AUC-ROC to have a detailed explanation. To achieve robustness, 5-fold cross-validation of training, 95% confidence intervals (CIs) and validation sets was performed. The discrimination of the classifiers was measured by the area under the curve (AUC) and the ROC plots were constructed to demonstrate how the model stood up against shallower classifiers.

Experimental results of the developed FuzzyFed-CNN model for the detection of AD are segmented into three categories: simulated, comparative, and statistical results. For the simulated results, multimodal MRI samples from the OASIS and ADNI datasets are plotted to reflect differing cognitive states. These data offer baseline input toward training and testing deep learning models. Interpretability outputs, such as heatmaps, show that the model identifies neurologically appropriate brain areas, which increases clinical confidence.

### 4.1 Ablation study

To isolate the contribution of each component for the proposed framework, we conducted an ablation study using four setups:

- CNN-only - monomodal MRI feature extraction.
- CNN + Metadata (MLP) - MRI features and structured clinical metadata fed into a multi-layer perceptron.
- CNN + Fuzzy Features - MRI features are fused with fuzzy-rule-based clinical features.
- Full FuzzyFed-CNN - multimodal combination of CNN and fuzzy features in the FL framework.

Table 5 shows the incremental improvements by multimodal integration, fuzzy inference, and federated optimization. We find that the CNN only model establishes a baseline level of performance, while the integration of metadata, fuzzy reasoning, and federated learning progressively improves diagnostic effectiveness and that adding metadata can provide sensitivity gains. Further, the addition of fuzzy features makes both accuracy and interpretability even better. The complete FuzzyFed-CNN model provides the best performance in all metrics, validating that all components have a meaningful role in the robustness of diagnosis. Comparative evaluation against MobileNet, DenseNet, EfficeintNet and ResNet confirmed that FuzzyFed-CNN consistently outperformed baselines across all metrics.

**Table 5:** Performance comparison with ablation study, statistical significance, and baseline models.

Model variant	Accuracy % (95% CI)	Sensitivity % (95% CI)	Specificity % (95% CI)	F1-Score % (95% CI)	*p*-value vs FuzzyFed	Cohen’s d	Outcome
CNN-only	88.4 (±1.5)	86.9 (±1.6)	87.5 (±1.8)	87.1 (±1.7)	<0.001	1.25	Statistically significant difference
CNN + Metadata (MLP)	91.2 (±1.3)	90.3 (±1.4)	91.0 (±1.5)	90.7 (±1.6)	<0.001	1.10	Significant
CNN + Fuzzy Features	94.8 (±1.2)	94.1 (±1.2)	95.0 (±1.3)	94.3 (±1.3)	0.004	0.65	Significant
FuzzyFed-CNN	**97.7 (±0.9)**	**98.0 (±1.0)**	**99.0 (±0.8)**	**98.0 (±0.9)**	—	—	Best performing model
MobileNet	80.5 (±2.8)	88.0 (±2.5)	85.0 (±2.6)	86.7 (±2.6)	<0.001	1.70	Significant
ResNet	82.0 (±2.6)	83.0 (±2.7)	83.5 (±2.5)	83.2 (±2.6)	<0.001	1.60	Significant
DenseNet	93.1(±2.1)	92.8(±2.6)	93(±2.1)	92.1(±2.0)	<0.001	1.62	Significant
EfficeintNet	96.5(±1.1)	95(±2.2)	97(±1.5)	97(±1.1)	<0.001	1.65	Significant

Bold values indicate the best performance achieved for each evaluation metric (FuzzyFed-CNN, the proposed model).

All reported values represent the mean performance obtained through five-fold cross-validation, and confidence intervals correspond to the 95% confidence level. Accuracy, sensitivity, specificity, and F1 are given as percentages with a 95 percent confidence interval (CI). The *p*-value column is a result of two-sample t-tests between each model and FuzzyFed-CNN. Effect size = Cohen’s d. Each of the baseline models had been optimized on a grid search with hyperparameters before assessment. These findings confirm that fuzzy inference provides substantial benefits beyond simple metadata integration, while federated training further boosts generalization across heterogeneous data sources.

The ablation experiment shows the contribution of each individual component within the framework. First, the performance of the imaging technique is used as the baseline, where only the CNN model is considered. Second, the CNN + Metadata model examines the performance after adding the structured medical data, but not through fuzzy reasoning. Third, the CNN + Fuzzy Features model tests the effect of uncertainty-driven reasoning in the clinical process. Last, the full framework FuzzyFed-CNN measures the performance of all three components working together.

### 4.2 Robustness checks and statistical analysis

In order to increase the confidence that the presented results are reliable, the experiments have been assessed with five-fold cross-validation and repeated five times independently from each other. The performance measures are shown in the form of means with 95% confidence intervals. Normality of distributions was confirmed prior to testing for significance by employing the Shapiro–Wilk test, whereas the assumption of equal variances was validated using Levene’s test. Both assumptions of normality and variance homogeneity have held true for all measures examined. As a result, two-sided independent samples t-tests were run to statistically compare the FuzzyFed-CNN model against other models. Effect sizes measured by Cohen’s d have also been reported together with *p*-values. Furthermore, Bonferroni correction was employed for multiple comparisons to minimize false-positive findings and enhance the robustness of the reported statistical significance results. It has been found that FuzzyFed-CNN statistically significantly improves upon CNN-only and CNN + MLP (*p* < 0.001) and improves significantly upon CNN + Fuzzy Features (*p* = 0.004). The range of Cohen’s d effect sizes is 0.65 (moderate), CNN + Fuzzy to FuzzyFed, and more than 1.0 (large) between CNN and CNN + MLP, which is practically significant rather than statistically significant. Top models have small confidence intervals (e.g., The confidence intervals for FuzzyFed-CNN were narrow, indicating stable performance across folds). To justify the selection of the CNN backbone, additional experiments were conducted using MobileNet, ResNet50, DenseNet121, and EfficientNet-B0 under identical experimental conditions, additional experiments were conducted with mobile net, resnet 50, testnet and efficient net. Identical experimental conditions were presented to all the algorithms. The deeper architecture demonstrated competitive performance, however the boost CNN based fuzzy fed framework achieved better overall diagnostic performance. Which means that they are consistent across the folds. All statistical tests were two-tailed and used *α* = 0.05. We also have corrected multiple comparisons using the Bonferroni approach to post-hoc analyses where the correction was important (Aggregated metrics that can be seen in Table 5, and class-wise evaluation can be seen in Table 6). Figure 5 shows samples from a multimodal dataset for the detection of AD, fusing scans from OASIS and ADNI data. The data are commonly used in neuroimaging research to facilitate the training of machine learning models for early diagnosis of Alzheimer’s. The first row contains structural MRI scans from the OASIS dataset that are annotated based on clinical dementia ratings (CDR) like “mild,” “very mild,” and “non-demented.” Scans typically are grayscale T1-weighted images of structural brain changes of neurodegeneration. The bottom row displays color-enhanced MRI slices of the ADNI dataset with the label normal or cognitively impaired. The pictures can also demonstrate false-color mapping and intensity normalization as preprocessing algorithms. It may help in visual analysis and computer analysis. Using a multimodal dataset increases the strength and generalizability of deep learning models through various representations of data. Variability in imaging protocols, demographic diversity, and disease staging among datasets supports in developing a richer diagnostic instrument. The following figure illustrates the complementary strengths of OASIS and ADNI in capturing different levels of cognitive impairment. It supports automated detection and monitoring of AD progression.

Figure 6 is the Receiver Operating Characteristic (ROC) curve plot to detect AD. ROC curve is a graphical representation of a classifier. The representation performance is done by plotting True Positive Rate (TPR) versus False Positive Rate (FPR) at various levels of thresholds. The diagonal dashed line represents a random classifier (AUC = 0.5), which serves as a baseline to measure performance. A model that performs would bow toward the top-left corner curve, which represents higher sensitivity and fewer false positives.

Curves are shown for FuzzyFed-CNN, MobileNet, DenseNet, EfficientNet and ResNet. FuzzyFed-CNN achieves the highest AUC, demonstrating superior diagnostic ability compared to baseline models.

Figure 7 shows the feature space representation of the clustering of simulated multimodal OASIS and ADNI data. Each dot is a subject, and the colour distinguishes between various classification categories (e.g., among classes of diagnosis or feature groups). UMAP aptly portrays global and regional structure; it allows seeing how subjects become separated into geographical regions. The upper cluster indicates that they are concentrated as a coherent group, whereas the lower region is more diffuse, indicating the lack of homogeneity. This is indicative of the fact that Uniform Manifold Approximation and Projection (UMAP) preserves fine-grained relationships between modalities, which may be useful to distinguish between disease subtypes. This representation is a testimony of how UMAP can manage a high-dimensional multi-encoded neuroimaging data into a meaningful two-dimensional topology.

Figure 8 shows the t-SNE feature space representation draws the similar multi modal OASIS and ADNI data as in projection in 2 dimensions. Each consequent point represents a subject, and its colour depends on the membership of the classes. t-SNE focuses on local neighborhood conservation and thus, subjects that have similar characteristics are clustered. The distribution is more evenly partitioned into classes than UMAP, especially some diagnostic categories, where classification boundaries become more fragmented. This indicates that t-SNE is useful to indicate local similarity, reflecting some differences in disease-related features. The figure shows the effectiveness of t-distributed Stochastic Neighbour Embedding (t-SNE) in revealing nonlinear topologies and intra-class variance in multimodal neuroimaging data with the potential to help explore AD development.

Comparative performance (see Figure 9) highlights the strengths and weaknesses of the three models applied to multimodal OASIS and ADNI data. FuzzyFed-CNN model is much better than the rest and has a remarkable accuracy of 97.7, 98.0% sensitivity, 99.0% specificity, and 98.0% F1-score clearly outperforming MobileNet (accuracy 80.5%, F1-score 85%) and ResNet (accuracy 82%, F1-score 83.5%). Precision values for MobileNet (85.4%) and ResNet (83.4%) were also significantly lower. This means that it is strong in identifying the right classification of Alzheimer’s and related conditions (Benyahia et al., 2024). In comparison, MobileNet and ResNet yield low accuracy of 88 and 83%, respectively. They have very low F1 scores and recall values, indicating poor generalization and asymmetrical predictions. Altogether, the FuzzyFed-CNN proves to be notably better and more credible than regular deep models.

Performance comparison of FuzzyFed-CNN and baseline models based on Accuracy, Precision, and F1-Score. The proposed model achieved 97.7, 98, and 98%, respectively, with an additional specificity of 99%, demonstrating superior diagnostic capability.

The values of sensitivity and specificity in Table 6 indicate the unstable performance of the currently available models, MobileNet and ResNet, across the various classes of AD (Radhika and Chintala, 2025). The sensitivity of MobileNet is also extremely diverse, reaching 1.0 with OASIS-Mild and decreasing to 0.0 with ADNI-CN and OASIS-Moderate, which is a sign that some categories are harder to detect. Specificity, however, is still fairly high, ranging from 0.8 to 1.0 in most classes, which implies fewer false positives. ResNet is a little more sensitive to ADNI-CI (0.8571) and OASIS-Very_mild (1.0), and not at all sensitive to ADNI-AD, OASIS-Mild, and OASIS-Moderate (Munipalli and Annepu, 2024). DenseNet and efficientNet shows improved class wise sensitivity and specificity as compared with MobileNet and Resnet. Efficient net achieved the most balanced last level performance due to compound skilling strategy. However, models demonstrate disturbed class-wise detection, which is a weakness in comparison with FuzzyFed-CNN.

**Table 6:** Class-wise sensitivity and specificity (MobileNet vs. ResNet vs. DenseNet *Vs* EfficientNet).

Dataset	Class	MobileNet sensitivity	MobileNet specificity	ResNet sensitivity	ResNet specificity	DenseNet sensitivity	DenseNet specificity	EfficientNet sensitivity	EfficientNet specificity
ADNI	CN	0.0000	1.0000	0.3333	0.9412	0.6667	0.9524	0.8571	0.9731
	CI	0.2857	0.9231	0.8571	0.6923	0.9000	0.8571	0.9444	0.9167
	AD	0.5000	0.5556	0.0000	1.0000	0.7500	0.8889	0.8750	0.9444
OASIS	Non_Demented	0.6000	1.0000	0.0000	1.0000	0.8333	0.9524	0.9231	0.9737
	Very_mild	0.5000	1.0000	1.0000	0.6667	0.8750	0.8333	0.9444	0.9167
	Mild	1.0000	0.8947	0.0000	1.0000	0.9091	0.9474	0.9524	0.9737
	Moderate	0.0000	0.9500	0.0000	1.0000	0.6667	0.9600	0.8571	0.9800

The confusion matrix gives a class-wise visualization of the prediction results and identifies the classes for which there may be misclassifications. It must be noted that the performance measures of accuracy, sensitivity, specificity, and F1-score are determined on the basis of aggregate results achieved after running five-fold cross-validation and are thus macro-average performance metrics. Hence, some misclassification at the class level cannot be considered as poor performance of the system as a whole. Most of the misclassification occurs between medically adjacent categories such as CN and MCI, AD, where feature overlap is possible. Nevertheless, the number of correctly classified subjects is significantly larger than the misclassified ones, and consequently, the presence of misclassifications at the class level does not affect the high diagnostic ability attained using the FuzzyFed-CNN architecture.

Table 7 confusion matrix identifies the results of the classification in the ADNI and OASIS datasets. It is visualized in Figure 10. In the cases of ADNI, cognitive normal (CN), CI, and AD, there is significant misclassification between ADNI-CN and ADNI-CI (166 cases and 80 cases, respectively), and ADNI-AD and ADNI-CN (166 cases and 80 cases, respectively). OASIS data demonstrates a greater within-class recognition, particularly in Mild and Very Mild dementia, although there is a significant amount of confusion between OASIS-Mild and OASIS-Moderate (583 and 76 cases respectively). Data imbalance or model bias was observed in the identification of Non-Demented OASIS subjects. Also, other cases are confluent with OASIS-Mild and Moderate. In general, the model is weak with the ADNI groups and effective with the OASIS dementia classification.

**Table 7:** Confusion matrix for the proposed model on the OASIS and ADNI datasets.

Dataset & class		ADNI			OASIS			
		CN	CI	AD	Non demented	Very mild	Mild	Moderate
ADNI	CN	28	166	92	5	0	0	0
	CI	67	265	193	13	0	0	0
	AD	21	80	120	2	0	0	0
OASIS	Non demented	0	0	0	0	0	0	0
	Very_mild	0	0	0	189	0	328	53
	Mild	0	0	0	8	0	524	76
	Moderate	0	0	0	0	0	583	28

The test data showing correctly and incorrectly classified samples across diagnostic categories. Axis labels and color gradients indicate class distribution and misclassification intensity. To help understand how the confusion matrix relates to the performance metrics described, overall accuracy, sensitivity, specificity, and the F1-score, are calculated as macro-averaged metrics across all classes, using five-fold cross-validation. While the confusion matrix shows all classes predictions, the performance metrics described in the end show the averages across all the folds and independent runs. Although there are some misclassifications, specifically in the clinically adjacent classes of CN and CI, AD the dominating diagonal elements show that the samples that are correctly classified overwhelmingly outnumber the samples that are misclassified. This accounts for the overall high performance metrics that are reported. In addition, the class-wise variability of the metrics reported is expected in multi-class neurological diagnosis that is due to the pathological characteristic overlap among the stages of the diseases.

#### 4.2.1 Statistical validation approach

In order to increase the credibility of the obtained results, each experiment was validated using the procedure of five-fold cross-validation, and eachs experiment was run independently several times. The performance values are reported using mean values with 95% confidence intervals. Prior to conducting statistical significance tests, the assumptions used for the tests were examined, namely the normal distribution and equal variance of data were confirmed through Shapiro–Wilk and Levene’s tests, respectively. Based on the results of the above-mentioned tests, two-tailed statistical significance tests have been performed for the proposed fuzzy-based CNN approach compared to the other approaches. The concept of practical significance is evaluated using the calculation of effect sizes (Cohen’s d). In order to reduce the risk of finding false positive results due to multiple testing, Bonferroni correction was applied whenever it was necessary.s.

### 4.3 Computational cost analysis

FuzzyFed-CNN required ~2.2 min of local training per client per round and converged in ~100 rounds (~3.5 h total). Communication overhead was moderate (~120 MB per round per client). In contrast, MobileNet (~0.4 min/round) and ResNet (~0.7 min/round), DenseNet (~1.1 min/round), and EfficientNet (~0.9 min/round) were faster but they also had significantly less accurate. Thus, the additional cost of FuzzyFed-CNN is justified by its superior diagnostic reliability (Alruily et al., 2025). The network latency was not explicitly measured in this simulation but is acknowledged as a practical constraint for real hospital deployments, which will be evaluated in future work.

### 4.4 Final implementation output

The last part of the FuzzyFed-CNN model implementation proves the efficiency and explainability of the suggested architecture. Training the model on multimodal datasets consisting of MRI and structured clinical information of the OASIS-3 and ADNI, offered under a FL architecture with fuzzy reasoning, the model was trained to guarantee privacy-preserving, yet explainable performance.

Figure 11 presents the evolution of training and validation accuracy in 100 rounds of federated communication of the FuzzyFed-CNN model. Accuracy increases gradually up to about 97 percent, which means that it is a stable model and learning occurs throughout in distributed clients.

Training and validation accuracy curves show a close correspondence during the process, which leads to the fact that the model is well-generalized and does not overfit. The global model effectively consolidates the information of all clients at a given FedAvg algorithm, resulting in a total accuracy of 97.7% at convergence.

Figure 12 shows that the training and validation loss decrease with increasing epochs. The loss curves of both loss curves have a smooth exponential decay, which indicates that it converges steadily, and the client drift is minimal when performing FL.

The training loss falls sharply within the first 30 epochs and levels off after 80 epochs, whereas the validation loss is on the same path and stabilizes at around 0.12. This gradual loss curve validates the fact that the FuzzyFed-CNN model is effective in reducing classification error without affecting federated synchronization and local training consistency.

Figure 13 shows a Grad-CAM heatmap of the FuzzyFed-CNN model, which indicates the most significant areas of an MRI brain slice that are applied to classify the disease as Alzheimer’s. The red and yellow activation zones are associated with the hippocampal and cortical areas, which are important biomarkers to diagnose Alzheimer’s. This visualization illustrates the XAI element of the FuzzyFed-CNN framework. The model can provide information on the contribution of some regions of the brain to its predictions by overlaying gradient-based activation maps on MRI scans. The targeted area of the hippocampus and cortex is associated with clinical evidence, thus enhancing the credibility and openness of the model decision-making.

## 5 Discussion

The results confirm the superior performance of the proposed FuzzyFed-CNN model in AD identification according to various evaluation metrics. Through the combination of CNNs for image processing with fuzzy logic-based clinical features within a FL framework. Comparative analysis against baseline models highlights the limitations of traditional approaches. MobileNet and ResNet models do not come close to the level of comprehension provided by the addition of structured clinical data. To further justify the selection of convolution neural network is a backbone architecture, additional experiments were conducted by considering the deep models including MobileNet, ResNet, DenseNet. The main thing that is kept constant is experimental settings. The performance of deep architecture is competitive, however the proposed fuzzy fed CNN achieved highest performance. These models perform poorly and erratically on both sensitivity and F1 score, and are hence less optimal for very high-complexity and heterogeneous clinical datasets. The above t-SNE and UMAP visualizations reveal the feature space of the OASIS and ADNI datasets in the FuzzyFed-CNN model. The two methods are potent dimensionality reduction procedures that project high-dimensional feature representations into two dimensions, so that clustering and class separability can be more readily interpreted. The t-SNE visualization reveals well-discernible cluster structures with apparent class separations, even though there is still some overlapping among the ADNI-CN, ADNI-CI, and ADNI-AD groups. This reflects that the earlier development of cognitive impairment may itself be a disease-common, feature-similar phenomenon. A different view in the UMAP shows much more condensed groupings with sharp edges on some classes, notably in the OASIS subgroups. The feature of locally and globally preserving the structure identifies the power of FuzzyFed-CNN in acquiring the discriminative features. Together, these visualizations confirm that the model effectively distinguishes between dementia stages, supporting its high accuracy. The class separability has been improved and was observed in both t-SNE and UMAP visualizations. It further validates the discriminative features-learning capability of the proposed model. The fact that FuzzyFed-CNN was able to outperform the baseline models can be explained by the fact that the former incorporated the complementary data modalities. Although CNN-only models present the opportunity to discover structural brain changes based on MRI images, they fail to pick up significant contextual details. This gap is not filled by a fuzzy inference system, which models uncertainty and adds clinical conditions, including age, MMSE scores, and genetic risk. The role of fuzzy inference in the proposed model is not limiting to classification accuracy however it contributes toward uncertainty modeling, soft decision making and enhanced interpretability. The multimodal fusion as such allows the model to generalize more across the heterogeneous patients, and this contributes to higher sensitivity and specificity. An ablation-oriented comparison between different models further demonstrates the performance improvement that is achieved with multimodal fusion, fuzzy reasoning and Federated learning. Conversely, Support Vector Machine (SVM), Decision Tree, and Random Forest all have poorer performance because they lack nonlinearity when it comes to capturing nonlinear relationships between variables in multimodal data. The other relevant benefit is the FL framework. FuzzyFed-CNN can be used to gain the advantages of data variety, but without the impact on the privacy of the patients that centralization implies by relying on highly diverse, decentralized datasets belonging to different institutions. Such diversity limits overfitting and leads to more robustness than centralized models trained on smaller, homogeneous data. The weaknesses of the current investigation are the use of publicly available datasets, which do not entirely represent clinical diversity in real hospitals. The existing model is restricted to MRI and only generic clinical characteristics, whereas other useful biomarkers, including those of PET scans, blood-based biomarkers, or longitudinal natural history data, were excluded. Further, federated training adds an overhead of communications, making it a potential bottleneck on large-scale deployments. Future plans include a future expansion of FuzzyFed-CNN to more modalities, including PET imaging and longitudinal clinical records, the application of more complex federated optimization algorithms to deal with non-IID data as well as an evaluation of the framework in real-life hospital collaborations. Trust can also be heightened by spreading explainability to other methods, such as a multi-layer model that can cover higher explanations than those introduced by Gradient-weighted Class Activation Mapping (Grad-CAM).

Emphasized clinical trust and doctor usability:

- Fuzzy rules provide human-readable reasoning.
- Grad-CAM heatmaps highlight hippocampal/cortical biomarkers.
- Multimodal fusion mimics how doctors synthesize info.

### 5.1 Limitations

While the proposed FuzzyFed-CNN framework shows great diagnostic accuracy and interpretability, a few limitations should be considered to provide transparency.

Dataset imbalance: The model was trained using a mixture of OASIS and ADNI datasets, which are not perfectly balanced in terms of sample size, distribution of disease stages, and demographic composition. For example, ADNI generated more cognitively normal and AD subjects than OASIS, which instead gave more mild and very mild dementia cases. This imbalance can have led to bias in classification performance by improving certain groups’ recognition performance.

Computational overhead: The sharp increase in the computational complexity of implementing fuzzy inference in a FL pipeline. Further, the fuzzy rule evaluation and defuzzification stages can increase training times relative to CNNs-only baselines. While this is still possible on modern GPUs, for resource-limited institutions, this overhead can restrict scalability.

Scalability across institutions: Peer-to-peer FL addresses the privacy issue, but hospital deployments in the wild are heterogeneous in client environments. Large heterogeneity: FuzzyFed-CNN scalability could be affected by the large variability of data quantity, availability of modalities, and network reliability. Specifically, if some institutions provide very small datasets as compared with others, then the global model may be biased toward the sites.

Generalisability: The study was carried out on publicly available datasets with standard preprocessing. Real clinical settings lead to noisier, incomplete, and less standardized data. Under such conditions, performance is yet to be proven. Detailed bandwidth utilization and communication cost measurements were not explicitly evaluated in the current study and will be investigated in future large-scale clinical deployments.

### 5.2 Future work

Many directions can expand the current study and promote the clinical applicability of FuzzyFed-CNN.

Integration of new modalities: In addition to MRI and curated phenotypic and demographic data, future work will include positron emission tomography (PET) imaging, blood-based biomarkers, and genetic data (e.g., APOE4 status). Combining these modalities may further enhance early-stage diagnosis of AD and a more complete picture of the progression of the disease.

Advanced federated optimization: Our current experiments used the standard FedAvg algorithm, which might be restricted by highly heterogeneous (non-IID) client distributions. We will test state-of-the-art federated optimization methods like FedProx and FedDyn that are tailored to deal with statistical heterogeneity and enhance convergence for the multimodal case.

Clinician-in-the-loop rule refinement: The FIS represents medical reasoning through predefined rules, but in subsequent versions, the system might also take clinician feedback during real-time operation. A good example of such refinement of an adaptive rule is improved adaptation based on clinical knowledge, which would increase trust and interpretability.

Scalability to real-world deployments: The framework will need to be expanded to multi-institutional hospital networks, dealing with bandwidth limitations, sporadic contributions, and heterogeneous hardware. Functional studies under these conditions, including real-time hospital workflows, will be necessary to ensure robustness and feasibility for clinical use.

Extended explainability: While Grad-CAM visualizations are informative, they should be supplemented with other explainability methods (e.g., SHAP, LIME, or counterfactual reasoning) to enhance explainability and trust from clinicians, particularly in multimodal data fusion scenarios.

### 5.3 Ethical considerations

Ethical concerns regarding privacy, consent, and control of information on patients taking part in medical AI studies are significant. This was studied by using publicly available data (OASIS and ADNI), which were approved by the informed patient consent and are also ethically approved in terms of research. To make it practical, use in the real world would require clear consent of the patient, to guarantee transparency and confidence in the methods of usage of data in model training cooperation. The FL scheme has a direct contribution to ethical AI practices since raw data is not allowed to be transferred between institutions. Sensitive patient data are not shared; only model updates are shared, so risks of data leakage or unauthorized access are lower. The practice fits the requirements of international laws (like HIPAA and GDPR), as well as motivating the development of cross-institutional cooperation without overstepping on privacy. Also, explainability methods such as Grad-CAM increase ethical transparency by enabling clinicians to ensure that outputs have been made on meaningful medical characteristics as opposed to spurious correlations. In general, the FuzzyFed-CNN model is an important example of how privacy techniques and interpretable results can be used to build ethical AI in medical care to strike the right balance between innovation and patient rights and trust.

Even though the framework developed here performed better than the chosen benchmarks, it should be noted that no particular architecture can claim to be the best solution for every problem of medical diagnosis. Results can differ due to various factors, including but not limited to the properties of the dataset itself, heterogeneity of institutions, imaging techniques, as well as configuration of the training process. While the presented model showed promising results, future research will explore ViT (Vision Transformer), Swin Transformer, hybrid CNN-transformer architecture models, and federated learning optimization algorithms like FedProx and FedDyn. PET and CT scans would also be considered as additional inputs to augment multimodality for better generalization.

Studies conducted recently have shown that Vision Transformers (ViT), Swin Transformers, Graph Neural Networks (GNN), multi-modal transformers, and federated transformers can be successfully used to diagnose Alzheimer’s disease. The use of those methods helps improve the quality of global feature modeling and long-distance dependency learning. Nonetheless, they usually demand much larger training datasets, more computing power, and higher communication overhead than traditional methods. As for the proposed FuzzyFed-CNN, it is designed to strike a balance between accuracy, interpretability, privacy-preserving, and effectiveness. The choice of the chosen CNN backbone helped achieve a balance between the ability to extract features and applicability in healthcare institutions that might lack high computational capacity. While transformers are certainly an interesting direction, their application in the context of privacy-preserving multi-modal federated systems is still being explored. Therefore, future research will involve experiments with transformer and graph-based models on top of the chosen framework.

## 6 Conclusion

In this paper, the author presents FuzzyFed-CNN, a privacy preservation federation architecture, which combines CNN-based MRI features with fuzzy rule-based clinical reasoning. On an OASIS/ADNI mixed sample, the full FuzzyFed-CNN achieved an accuracy of 97.7% and F1-score of 98.0%, much higher than any baseline CNNs or classical deep models (*p* < 0.001). These findings, along with explainability outputs (Grad-CAM) and dimensionality reduction plots (UMAP/t-SNE), show that multimodal fusion and fuzzy reasoning in a federated model are capable of enhancing diagnostic accuracy and clinician trust and maintaining patient privacy. Heatmap visualizations also validated that the model effectively targeted clinically significant brain areas, providing insight and enabling interpretability. The t-SNE and UMAP visualizations both confirm that the proposed FuzzyFed-CNN model has a discriminative ability. The two approaches prove that the salient dimension space is able to capture the difference between control, mild cognitive impairment, and AD groups across both the OASIS and ADNI datasets. Although t-SNE emphasizes the local clustering tendencies, UMAP allows better visualization of group separation, preserving both local and global structures. The convergent validity between these visualizations and the classification performance measures attests to the validity of the model, which makes it useful in predicting the stage of dementia successfully. Future research will work on scaling up FuzzyFed-CNN to bigger, more diversified datasets, including the support of more modalities such as genetic and clinical information, and improved real-time provisioning to healthcare systems. The combination of explainable AI and lightweight optimization can enhance transparency, scalability, and the feasibility of dementia screening and monitoring. Altogether, the findings demonstrate that FuzzyFed-CNN is a scalable, interpretable, and privacy-preserving early Alzheimer detection method. This framework is highly promising in clinical practice and in terms of its future contribution to the development of diagnostic methods in neurodegenerative disease studies due to its technical novelty and clinical interpretability.

## Data Availability

The datasets analysed are publicly available: OASIS dataset: https://www.kaggle.com/datasets/ninadaithal/imagesoasis and ADNI sorted data: https://www.kaggle.com/datasets/summaiyamahmood/adni-sorted-data.
